# Comparing Quantitative Methods for Analyzing Sediment DNA Records of Cyanobacteria in Experimental and Reference Lakes

**DOI:** 10.3389/fmicb.2021.669910

**Published:** 2021-06-18

**Authors:** Hebah S. Mejbel, William Dodsworth, Alexandre Baud, Irene Gregory-Eaves, Frances R. Pick

**Affiliations:** ^1^Centre for Advanced Research in Environmental Genomics, Department of Biology, University of Ottawa, Ottawa, ON, Canada; ^2^Department of Biology, McGill University, Montréal, QC, Canada; ^3^Groupe de Recherche Interuniversitaire en Limnologie, Montréal, QC, Canada

**Keywords:** droplet digital PCR, high-throughput sequencing, metabarcoding, paleolimnology, sedDNA, targeted PCR

## Abstract

Sediment DNA (sedDNA) analyses are rapidly emerging as powerful tools for the reconstruction of environmental and evolutionary change. While there are an increasing number of studies using molecular genetic approaches to track changes over time, few studies have compared the coherence between quantitative polymerase chain reaction (PCR) methods and metabarcoding techniques. Primer specificity, bioinformatic analyses, and PCR inhibitors in sediments could affect the quantitative data obtained from these approaches. We compared the performance of droplet digital polymerase chain reaction (ddPCR) and high-throughput sequencing (HTS) for the quantification of target genes of cyanobacteria in lake sediments and tested whether the two techniques similarly reveal expected patterns through time. Absolute concentrations of cyanobacterial 16S rRNA genes were compared between ddPCR and HTS using dated sediment cores collected from two experimental (Lake 227, fertilized since 1969 and Lake 223, acidified from 1976 to 1983) and two reference lakes (Lakes 224 and 442) in the Experimental Lakes Area (ELA), Canada. Relative abundances of *Microcystis* 16S rRNA (MICR) genes were also compared between the two methods. Moderate to strong positive correlations were found between the molecular approaches among all four cores but results from ddPCR were more consistent with the known history of lake manipulations. A 100-fold increase in ddPCR estimates of cyanobacterial gene abundance beginning in ~1968 occurred in Lake 227, in keeping with experimental addition of nutrients and increase in planktonic cyanobacteria. In contrast, no significant rise in cyanobacterial abundance associated with lake fertilization was observed with HTS. Relative abundances of *Microcystis* between the two techniques showed moderate to strong levels of coherence in top intervals of the sediment cores. Both ddPCR and HTS approaches are suitable for sedDNA analysis, but studies aiming to quantify absolute abundances from complex environments should consider using ddPCR due to its high tolerance to PCR inhibitors.

## Introduction

Lake sediments act as natural archives through the accumulation and preservation of organic and inorganic matter arising from the lake as well as its watershed and airshed. Changes within lake ecosystems, whether natural or anthropogenic, are stored in sediment profiles and can provide long-term historical records of environmental change (Smol, 2008). Because of the general lack of long-term monitoring records, lake sediments can be used to help identify the drivers of environmental change, such as climate change or nutrient loading. Typically, paleolimnological studies rely on subfossils or distinct chemicals preserved in sediment as proxies of past environments (Smol, 2008). However, not all organisms preserve well in sediment or leave behind a distinct biomarker, making it difficult to understand and track long-term responses to environmental change. As an alternate and complementary method to classical paleolimnological proxies, various DNA-based methods have emerged (Domaizon et al., 2017).

The recent advances and application of molecular approaches using DNA archived in sediment provide for considerable expansion in this field, particularly for analyses of microbial communities. Quantitative PCR techniques have shown to be successful in quantifying specific targets from sediment DNA (sedDNA) including bacteria, viruses, and other microorganisms that lack morphological features (Coolen et al., 2006; Savichtcheva et al., 2011; Brazeau et al., 2013; Pal et al., 2015; Pilon et al., 2019). Other, community-wide analyses have also been proven to track and describe community dynamics through time (Monchamp et al., 2016, 2018; Legrand et al., 2017; Pilon et al., 2019). While there have been a few studies that have used a combination of molecular techniques on sedDNA, predominantly PCR-directed methods (Singh et al., 2017; Hamaguchi et al., 2018), there remains a lack of research directly examining the coherence and correlation between quantitative methods. This is of particular concern in the analysis of sediment samples where PCR inhibitors (such as humic acids) can be found at high levels, which can then interfere with the sensitivity of the technology used and/or produce false results (Rochelle et al., 1992; Savichtcheva et al., 2011; Albers et al., 2013). Although inhibition is an issue for all PCR methods, diluting samples can sometimes overcome these limitations, yet this is not always possible or suitable when calculating concentrations of low-level test samples. Alternate methods should be applied as some PCR techniques have been found to be more tolerant to inhibitors than others (Taylor et al., 2017, 2019; Wood et al., 2019).

Quantitative studies have mainly used quantitative real-time polymerase chain reaction (qPCR) to obtain relative abundances of target genes from environmental samples (Nathan et al., 2014; Pal et al., 2015; Pilon et al., 2019). Although qPCR does have advantages, the need to run samples in triplicate along with ensuring the assay shows reliable amplification can make it challenging to quantify targets from complex environments (Taylor et al., 2019) such as sediments. With the development of droplet digital polymerase chain reaction (ddPCR), these limitations are reduced. With ddPCR, water-oil emulsion chemistry is used to split the PCR reaction into 20,000 individual vessels, or droplets, before amplification, reducing the normalization and calibration issues of qPCR (Hindson et al., 2011; Taylor et al., 2017). Studies that have described the relationship between qPCR and ddPCR techniques using sediment samples found that ddPCR significantly improved both the sensitivity and the detection of low concentrations of target genes (Singh et al., 2017; Hamaguchi et al., 2018).

In addition to targeted molecular approaches, there has been a recent emergence of paleolimnological studies using metabarcoding techniques on sediment samples for community-wide analyses (Monchamp et al., 2016; Legrand et al., 2017; Pilon et al., 2019). High-throughput sequencing (HTS) provides for a cost-effective alternative to traditional paleolimnological methods by enabling simultaneous taxonomic identifications that probes sediment diversity, at times with increased specificity (Parducci et al., 2017; Tse et al., 2018). While there is still some uncertainty in interpretating the relative abundance data generated using metabarcoding approaches (Taberlet et al., 2018), some investigators are also exploring whether absolute read counts provide useful information (Lamb et al., 2018; Bylemans et al., 2019). Quantitative data using read counts obtained from metabarcoding has been suggested as an approach to monitor species interactions and distributions in support of management efforts (Hänfling et al., 2016; Ushio et al., 2018), but additional calibration is required to evaluate the quantitative performance of metabarcoding techniques. Some factors that can affect quantification data from HTS include the choice of reference database, primer biases that lead to unequal amplification (e.g., Hong et al., 2009; Shelton et al., 2016), and the bioinformatic pipeline chosen for analysis.

The performance between targeted and metabarcoding approaches using read counts has been evaluated in recent contemporary environmental DNA (eDNA) literature (Harper et al., 2018; Lamb et al., 2018; Bylemans et al., 2019). These studies found targeted PCR approaches were more sensitive with greater detection than eDNA metabarcoding methods. Although, others have found strong positive relationships between qPCR, ddPCR, and HTS technologies when using eDNA from seawater and biofouling samples (Wood et al., 2019). Quantitative outputs using absolute abundances from targeted PCR approaches (such as ddPCR) and read counts from metabarcoding approaches (such as HTS) has, to our knowledge, never been compared over timescales of decades using sedDNA. Furthermore, a greater amount of humic substances are often found in recent (or top) sediments (Calace et al., 2006; Hou et al., 2014) and these materials can affect the sensitivity of different genetic methods, and consequently the agreement among them. The physical and chemical changes of sediments through time (i.e., diagenesis) could also affect differentially how well the two methods amplify target genes.

In this study, we evaluated the coherence between ddPCR and HTS methods using DNA extracted from lake sediments at the Experimental Lakes Area (ELA). The ELA, located in Central Canada, has been the site of several long-term, ecosystem-level experimental manipulations (e.g., Schindler et al., 1990, 1996) and presents a unique opportunity to compare paleolimnological records across experimental and reference lakes. The latter have not been manipulated experimentally nor impacted by watershed land use change. However, reference lakes may still be subject to climatic and atmospheric changes or the occasional forest fire. Furthermore, long-term monitoring records are available for many ELA lakes which enables validation of sediment proxies (e.g., Leavitt and Findlay, 1994). Cyanobacteria were selected as the ideal test organisms for this study as lake manipulation at several ELA sites has led to significant changes in cyanobacterial abundance and composition. We chose Lake 227 as it has experienced continued experimental fertilization since 1969 with a well-documented and concomitant increase in the abundance and dominance of cyanobacteria (Schindler, 1974; Anderson et al., 1987; Schindler et al., 1990; Findlay and Kasian, 1996). Cyanobacterial dynamics have also been recorded in a sediment core from Lake 227 through pigment analysis (Leavitt and Findlay, 1994). Lake 223 is another experimental lake where sulfuric acid was applied to the lake between 1976 until 1983 to simulate the effects of acid precipitation (Schindler et al., 1980; Findlay and Kasian, 1986). In Lake 223, phytoplankton biomass increased under acidification, but since ~1994, the lake has largely recovered to pre-manipulation conditions (Findlay and Kasian, 1996). Here, our objectives were to (1) measure the level of agreement between absolute cyanobacteria counts derived from HTS against absolute concentrations derived from ddPCR, (2) compare relative abundances of *Microcystis* (a common genus of bloom-forming cyanobacteria) between the two methods, and (3) analyze the relationship between ddPCR and HTS from top and bottom sediments. We tested the hypothesis that ddPCR and HTS reflect similar patterns within each time series but that the level of coherence between the two methods would be weaker in top sediments as a result of potential organic matter interference.

## Materials and Methods

### Study Sites

Four lakes at ELA were used to determine the coherence between ddPCR analyses of gene copy numbers and HTS analyses of cyanobacterial gene targets. Lake 227 (L227) is an experimentally manipulated lake where nitrogen and phosphorus were added to the lake between 1969 and 1989. From 1990 onwards, only phosphorus has been continuously added (Schindler et al., 2008). Experimentation on Lake 223 (L223) began in 1976 when H_2_SO_4_ was added into the lake to drop the pH from 6.7 to 5.1 (Schindler et al., 1980; Cruikshank, 1984; Findlay and Kasian, 1986). Experimentation ended in 1983 to allow pH levels to naturally recover (Findlay and Kasian, 1990). Lakes 224 (L224) and 442 (L442) have been not been subjected to eutrophication nor acidification experiments and have served as reference lakes to manipulated lakes in previous studies (Findlay and Kasian, 1996; Higgins et al., 2018; Lamothe et al., 2018). L224 was subject to short radioisotope experiments in 1976 (Hesslein et al., 1980a,b; Quay et al., 1980; Crusius and Anderson, 1995). A map of ELA, lake coordinates, morphology, and lake manipulation details are presented in Supplementary Data (Supplementary Figure 1, Supplementary Table 1).

### Sediment Collection and Sectioning

In March 2018, four sediment cores were collected from the maximum depths of lakes 227, 223, 224, and 442 using a 1.2 × 0.1 × 0.2-m aluminum freeze corer [collected by the International Institute for Sustainable Development–Experimental Lakes Area (IISD–ELA), Supplementary Table 2]. Because of the flocculent nature of sediments in L227, freeze cores are a preferred way to obtain sediment cores from this lake (Michael Paterson, IISD-ELA, personal communication) and the same device was therefore used for all the lakes. Freeze coring is advantageous in obtaining undisturbed sediment, unaffected by the degassing of gas bubbles occurring from a change in hydrostatic pressure (Verschuren, 2000). The corer was filled with crushed dry ice and methanol and lowered through a hole in the ice. After ~20 min, the freeze cores were brought to the surface and, using a putty scraper, the excess unfrozen sediment was removed. A hammer and chisel were used to free the edges of the frozen sediment core from the freeze corer. The cores were then wrapped in cellophane, packed with dry ice and shipped to McGill University (Montréal, Québec) for subsectioning in the laboratory of I. Gregory-Eaves (Department of Biology).

In a cool sterile room, a rotary tool was used to subsample the sediment slab into 3 cm working intervals. Sterilized ceramic knives were used to scratch off a very thin surface layer from the face of the 3 cm intervals, moving horizontally across the core surface. Using the rotary tool, and following the line indicated by a laser and plastic meterstick, 0.5 cm subsections were created by making a shallow incision in the sediment. A ceramic knife was then inserted horizontally across the incision and tapped on using a sterilized hockey puck to cleave the slice. The workspace, ceramic knives and rotary tool blades were sterilized with bleach, rinsed with deionized water, and dried with KimWipes^®^ between each subsection. This process was continued until each 3 cm interval blocks along the core were subsectioned. Every other 0.5 cm subsection was collected for molecular analyses while remaining samples were collected for other applications. Samples were stored in 5 ml sterile screwcap centrifuge tubes (VWR) then frozen at −20°C. All subsequent molecular analysis steps (extractions, optimization, and PCRs) were conducted in a separate and sterile part of the Core Molecular Biology and Genomics Laboratory at the University of Ottawa (Ottawa, Ontario).

### DNA Extraction

Sediment DNA was extracted using the DNeasy PowerSoil Kit according to the manufacturer's instructions (Qiagen, Germany). Minor modifications were made to the protocol. Lake sediment samples, ranging from 0.5 to 0.7 g, were added to emptied PowerBead tubes. If DNA yield and quality did not meet standards, wet sediment weight was increased to 1 g. Subsection intervals from L224 and L442 were washed twice, and intervals from L227 and L223 were washed three times using 250 μl of a wash buffer found to improve DNA quality and quantity yield by removing humic compounds and divalent cations (Zhou et al., 1996; Poulain et al., 2015). Samples were vortexed for 3 min at maximum speed on a Vortex-Genie 2 (Scientific Industries Inc., USA), followed by a centrifuging step at 3,000 r.p.m. for 3 min. After discarding the supernatant from the final washing step, the original PowerBead tube contents (glass beads, salts, and buffers) were re-added to the tubes. The extraction kit protocol was then carried out, but centrifuge time was increased to 1 min at each centrifuge step. The purity of the DNA was measured using a NanoDrop^TM^ 2000 Spectrophotometer (Thermo Scientific, USA) and estimated based on the ratios of absorbance at 260 and 280 nm, and at 260 and 230 nm. DNA was considered pure if the ratios were at or around 1.8. Lower ratios indicate some protein contamination (if 260:280 ratios are low) or organic contamination (if 260:230 ratios are low) (Yeates et al., 1997). DNA quantity was measured through a Quant-iT dsDNA assay kit (Invitrogen). Extractions were repeated with a decreased elution volume of 50 μl if low DNA yield and purity persisted. Final DNA extraction solutions were stored at −20°C for downstream applications and then −80°C for long-term storage.

### Sediment Dating

For the creation of the dating profiles, sediment samples (*n* = 12–15) were homogenized, freeze-dried at −50°C, and their water content measured. Samples were then placed in an Ortec High Purity Germanium Gamma Spectrophotometer (Model GWL-120230, Oak Ridge, TN, USA) at the Laboratory for the Analysis of Natural and Synthetic Environmental Toxins (LANSET) core facility to measure ^210^Pb, ^137^Cs, and ^226^Ra activity. Constant Rate of Supply (CRS) models were chosen for all cores and cross-calibrated against the ^137^Cs peak (Blais et al., 1995). Depth-age profiles are presented in Supplementary Figure 2. The peak from the ^137^Cs was used as an independent marker and estimated to occur in 1963, when maximum nuclear weapon testing was in effect before atmospheric testing became prohibited.

Additional measures were taken with the L227 record to ensure the robustness of the chronology because ^226^Ra was added to L227 in 1970 (Emerson and Hesslein, 1973), which is mobile in sediment and degrades to ^210^Pb, potentially hindering accurate dating profiles. As a first step, we calculated ^210^Pb excess activity through point-by-point subtractions of the supported activity from the total activity. To corroborate and confirm the ^210^Pb dating, annual lamination (i.e., varve) counting was also conducted based on a high-resolution photograph taken by the Itrax XRF cores scanner housed at L'Institut National de la Recherche Scientifique in Québec.

### Pre-droplet Digital PCR Optimization

Prior to running ddPCR, primers for each target gene were validated through qPCR (as suggested by Taylor et al., 2017). The cyanobacterial target gene chosen for analysis included cyanobacterial 16S rRNA (CYA; universal gene essential for DNA translation), as a proxy for the total abundance of cyanobacteria. The primer set chosen amplifies the V1–V3 regions of the 16S rRNA gene conserved in cyanobacterial species (Nübel et al., 1997; Rinta-Kanto et al., 2005). In addition, *Microcystis-*specific 16S rRNA (MICR) was also targeted to allow for testing the limits of the methods. *Microcystis* is a widely distributed and well-studied freshwater genus (Harke et al., 2016) that occurs in ELA lakes at low concentrations. Primers and associated sequences are listed in Table 1. All reactions were cycled in a Bio-Rad CFX96 Real-Time PCR Detection module and analyzed using Bio-Rad CFX Manager Software version 3.0. Each PCR mix contained 4 μl of 10x diluted pooled DNA, 0.2 μl of nuclease-free water, 0.4 μl of each primer (forward and reverse), and 5 μl of the 2x SsoFast/Sso Advanced EvaGreen Supermix, brought to a final volume of 10 μl. The samples were then loaded in 0.2 ml eight-strip tubes in triplicate, run alongside one positive and one negative control sample. For the positive controls, a sample volume of 4 μl of DNA extracted from pure cultures of the microcystin-producing *Microcystis aeruginosa* strain CPCC 300, was used (CPCC300, Canadian Phycological Culture Center, Canada). Nuclease-free water was used for the negative controls. The annealing temperature for each primer set was first optimized using a thermal gradient. The qPCR cycle program consisted of an initial preheating step of 95°C for 3 min, followed by 40 cycles of denaturation at 95°C for 10 s, annealing for 20 s, and extension at 72°C for 20 s. The melt curve protocol followed with 10 s at 95°C and then 5 s each at 0.5°C increments between 60 and 95°C.

**Table 1:** The primer sequences used in droplet digital PCR (ddPCR) and high-throughput sequencing (HTS), amplicon sizes, annealing temperatures, and dilution factors of target genes cyanobacterial 16S rRNA and *Microcystis* 16S rRNA.

**Gene**	**Primers**	**Sequence (5′-3′)**	**Amplicon size (bp)**	**Annealing *T* (°C)**	**Dilution factor**
*Microcystis* 16S rRNA (ddPCR)	MICR F^a^ MICR R^a^	GCC GCR AGG TGA AAM CTA A AAT CCA AAR ACC TTC CTC CC	247	56	10x
Cyanobacteria 16S rRNA (ddPCR)	CYA 108F^a^^,^^b^^,^ CYA 377R^a^^,^^c^	ACG GGT GAG TAA CRC GTR A CCA TGG CGG AAA ATT CCC C	269	56	32x
Cyanobacteria 16S rRNA (HTS)	CYA 359F^c^^,^^d^ CYA 809R^c^^,^^d^	GGG GAA TYT TCC GCA ATG GG GCT TCG GCA CGG CTC GGG TCG ATA	450	52	10x, 50x

a *Rinta-Kanto et al. (2005)*.

b *Urbach et al. (1992)*.

c *Nübel et al. (1997)*.

d *Jungblut et al. (2005)*.

The qPCR amplification products were then separated through 1.5% agarose gels at 100 V in 1x TAE buffer for ~30–45 min. Products were then visualized using an MBI Lab Equipment Fusion UV visualizer cabinet (Montreal-Biotech, Canada) and captured using Fusion Molecular Imaging Software.

### ddPCR

The ddPCR technology was used to amplify and quantify CYA and MICR. Prior to running ddPCR on the unknown samples, pooled DNA (containing 5 μl from each extraction) was used to optimize the reaction conditions through temperature and dilution gradients. Temperature gradients were performed to establish the optimal annealing temperature and dilution gradients were run to determine the optimal DNA dilution and avoid saturation of the ddPCR reaction. Annealing temperatures and dilution factors are listed in Table 1.

Each PCR reaction was prepared with 5 μl of extracted DNA template, 6.04 μl of DNase-free water, 0.23 μl of each primer, and 11.5 μl of EvaGreen ddPCR Supermix. The samples were transferred according to the manufacturer's instructions into a DG8 cartridge with 65 μl of the QX200Droplet Generation Oil for EvaGreen (BioRad: 186-4005) and converted into droplets using a BioRad QX200 Droplet Generator. After the droplets were generated, 40 μl of the samples were then transferred into a ddPCR 96-well plate. Plates were sealed with specialized foil using a PX1 Plate Sealer then placed into a C1000 Touch Thermocycler and amplified under the following conditions: 95°C for 5 min, followed by 40 cycles of 95°C for 30 s, annealing for 1 min, and 4°C for 5 min with a −2°C/s ramp rate. This was followed by a final step of 90°C for 5 min for stabilization. After PCR amplification, the 96-well plate was transferred into the QX200 Droplet reader for quantification. Quantification of gene copy number per μl was determined using the QuantaSoft Software from Bio-Rad. Samples were accepted if at least 13,000 droplets were created and amplified.

To determine the absolute abundance of each target gene per gram of sediment, concentrations were corrected for DNA template volume, sample volume (volume of transferred PCR reaction mix), elution volume, and dilution factor before normalizing to the mass of dry weight (Equation 1). Gene copy numbers were normalized per gram of dry weight because water content was higher in top intervals of the sediment cores. The range in water content (%) in top and bottom sections of each core is presented in Supplementary Table 3.

(1) $$\begin{array}{r} Absolute\;abundance=\frac{ddPCR\;concentration\;*\left(\frac{sample\;volume}{template\;volume}\right)*\;elution\;volume\;*\;dilution\;factor}{mass\;of\;dry\;sediment} \end{array}$$

Relative abundances of MICR derived from ddPCR were calculated using: (MICR abundance/CYA abundance) × 100. The limit of detection (LOD) was determined by calculating the limit of the blank (LOB) and adding it to 1.645 times the standard deviation (*SD*) of low analyte samples (Armbruster and Pry, 2008): LOD = LOB + 1.645(*SD*_lowanalytesamples_). The LOB was calculated using: mean_blanks_ + 1.645(*SD*_blanks_). Eight pooled DNA samples, diluted 100x for MICR and 1000x for CYA, were used to calculate the *SD* of the low analyte samples. The LOD was determined as 1.55 copies/μl (copies per microlitre of reaction) for MICR and 1.43 copies/μl for CYA. After carrying through with normalization, the LOD for the MICR target was calculated as ~1.4 * 10^5^ copies/g of dry sediment for L227 and ~2.8 * 10^5^ copies/g for lakes 223, 224, and 442. The LOD for CYA was ~4.1*10^5^/g for L227 and ~8.2 * 10^5^ copies/g for the other three lakes. These LODs are conservative estimates and values obtained using ddPCR were, at times, below the average LODs for this method.

### High-Throughput Sequencing

Library preparation and paired end sequencing were completed at Genome Quebec Innovation Centre (Montréal, Quebec). Specific CYA primers (Table 1), with attached overhang adaptors, were used to amplify a 450-nt-long fragment of the V3–V4 regions of the 16S rRNA gene for metabarcoding (Nübel et al., 1997; Jungblut et al., 2005; Pilon et al., 2019). The PCR amplification was prepared with 1 μl of DNA template diluted 10x (few samples required a 50x dilution), 5.512 μl of DNase-free water, 0.048 μl of each primer, 0.032 μl of Qiagen HotStarTaq DNA polymerase, 0.16 μl of dNTP mix, 0.4 μl of Roche DMSO, and 0.80 μl of Qiagen 10x Buffer with 15 mM MgCl_2_. The PCR cycle program consisted of heating at 96°C for 15 min, followed by 35 cycles of 96°C for 30 s, 52°C for 30 s, and 72°C for 60 s. This was followed by a final step of 72°C for 10 min. Barcodes were added to each sample through a second round of PCR. Amplicons were then quantified using Quant-iT™ PicoGreen^®^ dsDNA Assay Kit (Life Technologies). The library was generated by pooling equal volumes of each sample, subsequently cleaned up with sparQ PureMag beads (Quantabio) and quantified using Kapa Illumina GA with Revised Primers-SYBR Fast Universal Kit (Kapa Biosystems). The amplicon pool was loaded at a final concentration of 7 pM with a 15% PhiX spike to improve the unbalanced base composition. Sequencing was performed on an Illumina MiSeq PE300 platform using the Miseq Reagent Kit. Two nuclease-free water samples were included as negative controls.

### HTS Data Processing

Raw FASTQ files obtained from Genome Quebec were demultiplexed from within the MiSeq Illumina workflow. The 16S rRNA gene sequence data were then processed using the QIIME2 pipeline (v 2019.7; Bolyen et al., 2019). The cutadapt command line tool was used to trim the forward and reverse primer site of the merged reads. DADA2 denoise-single plugin was used to truncate and remove sequences with an average Phred quality score below 20, create the amplicon sequence variant (ASV) table with representative sequences and their frequency of occurrence, perform quality filtering, and remove chimeric sequences. The sequence variants were assigned using the q2-feature-classifier Naïve Bayes classifier after extracting the region of the 16S sequences most appropriate for the primers (359F/809R) used during sequencing. The classifier was trained on the SILVA 99% identity Full Seq OTU reference database (v 138) using the classify-sklearn command on the trained classifier. An additional classifier was produced and trained on the Greengenes 99% OTU reference database (v gg_13_8_99) for comparison. Amplicon sequence variants assigned to chloroplasts or non-cyanobacterial species were removed from further analysis using the taxa filter-table plugin. Cyanobacterial and *Microcystis* ASV counts were normalized to counts per gram of dry sediment similarly to ddPCR (Equation 1). Absolute cyanobacterial ASV counts were compared against absolute gene copy numbers derived from ddPCR to determine how well ASV counts track changes in abundance at the phylum level. Due to the compositional nature of the data, the coherence between ddPCR and HTS at the level of genus was only compared using relative abundances of *Microcystis*. Proportions were exported using the feature-table relative-frequency plugin and converted into percentages by multiplying by 100. Absolute ASV counts for *Microcystis* were compared between SILVA and Greengenes to assess the consistency of these databases at the genus level. A summary of the bioinformatics workflow is presented in Supplementary Figure 3. The negative controls had <0.1% of the average number of reads and were removed from analysis.

Here, the HTS LOD was estimated as one read (in expectation) per sample. For the absolute abundances, the LOD for both CYA and MICR was calculated as ~8.8 * 10^5^ copies/g for L227 and 1.8 * 10^6^ copies/g for lakes 223, 224, and 442.

### Statistical Analyses

All statistical analyses were conducted using R open source (Version 4.0.0) and RStudio software (version 1.2.5042). Absolute gene copy data were first verified for normal distribution through Shapiro-Wilk tests. Data were log or square root transformed to approach normal distribution and minimize heteroscedasticity of variance. Absolute abundances of CYA and MICR from ddPCR and HTS were plotted as a function of sediment depth. Proportions of MICR were also plotted as a function of sediment depth. Pearson's *r* and Kendall's τ (for L227; due to the lack of normality, even after log-transformation) correlation coefficients were calculated to evaluate the relationship between the two methods across the entire sediment core, top sediments, and bottom sediments. All ddPCR-derived MICR gene copy numbers that were below the corresponding LOD were handled by using the common approach of substituting those values with the LOD/2 (in line with guidance provided by the United States Environmental Protection Agency, 2006). For the HTS data, samples with sequence reads below the detection limit (i.e., had zero counts) were replaced by a conservative pseudo-count of 0.5. The use of LOD/2 has been found to be reliable in studies where much of the data are below the detection limit or where data are highly skewed (Glass and Gray, 2001). Relative abundances of MICR (as determined from ddPCR, SILVA, and Greengenes) were transformed by employing arcsine square root transformations. Temporal autocorrelation is an issue common in analyses of sediment records and environmental data in general and can potentially lead to the lack of independence among time points in the collected samples (Legendre, 1993; Taranu et al., 2018). To test for the effect of temporal autocorrelation on *p*-values, gene copy numbers were subjected to the autocorrelation function (ACF) in R. If both variables within the correlation were found to be temporally autocorrelated, the *p*-value was corrected by permuting by blocks using the *permute* packages in R. If either variable was not found temporally autocorrelated, the *p*-value was not corrected (Legendre and Legendre, 2012).

Using ^210^Pb dating profiles (see Supplementary Data—Supplementary Figure 2), top sediment samples for experimental lakes 227 and 223 were selected to correspond to and include the intervals associated with the approximate period of lake manipulation and all subsequent sediment deposition (1969–2018 for L227 and 1976–2018 for L223). Bottom sections chosen for reference lakes 224 and 442 were chosen to roughly parallel L223 bottom sections because sediment intervals corresponded to relatively similar timeframes in the pre-manipulation period (Supplementary Table 4). Paired *t-*tests were also performed to assess statistical differences in DNA content between recent samples (top sediments) and older samples (bottom sediments).

## Results

DNA was successfully extracted from all sections sampled from each lake core. The experimental lakes had more DNA in top sediments compared to the bottom sediments relative to the reference lakes (Table 2): for L227 the mean DNA concentration was 1.6X greater (*p*-value < 0.01) and for L223, 1.26X greater (*p*-value < 0.05). In contrast, there was no statistical difference in DNA content between top and bottom sections for L224 and L442 (*p*-values > 0.05 for both lakes). For all four cores, the mean DNA quality was high (260:280 ratio > 1.7). The bottom intervals of L227 and L223 contained DNA of higher purity (260:280 ratio > 1.8) in comparison to the top subsection intervals. Only the top sections from L227 had a mean ratio <1.7. All lakes showed very low 260:230 ratios.

**Table 2:** Comparison of mean DNA concentration (± *SE*) and purity between top and bottom subsection intervals.

**Lake**	**Core depth (cm)**	**Number of subsections**	**DNA (ng/g of dry sediment)**	**260:280 ratio**	**260:230 ratio**
227	Top (1–15.25)	11	4,102 (± 355)	1.62 (± 0.02)	1.15 (± 0.08)
	Bottom (16.25–51.75)	12	2,577 (± 255)	1.81 (± 0.02)	1.39 (± 0.08)
	Total core	23	3,306 (± 266)	1.72 (± 0.03)	1.28 (± 0.06)
223	Top (1–20.25)	15	2,870 (± 176)	1.74 (± 0.03)	1.41 (± 0.07)
	Bottom (21.75–41.75)	9	2,311 (± 152)	1.82 (± 0.07)	1.39 (± 0.08)
	Total core	24	2,660 (± 134)	1.77 (± 0.03)	1.40 (± 0.05)
224	Top (1–7.25)	9	4,662 (± 508)	1.86 (± 0.01)	1.28 (± 0.07)
	Bottom (16.25–32.25)	4	4,324 (± 414)	1.86 (± 0.02)	1.35 (± 0.05)
	Total core	13	4,558 (± 367)	1.86 (± 0.01)	1.29 (± 0.05)
442	Top (1–12.25)	8	4,183 (± 780)	1.90 (± 0.03)	1.33 (± 0.13)
	Bottom (13.25–39.75)	6	4,098 (± 754)	1.99 (± 0.08)	1.30 (± 0.10)
	Total core	14	4,146 (± 530)	1.93 (± 0.04)	1.32 (± 0.08)

**Figure 1:**
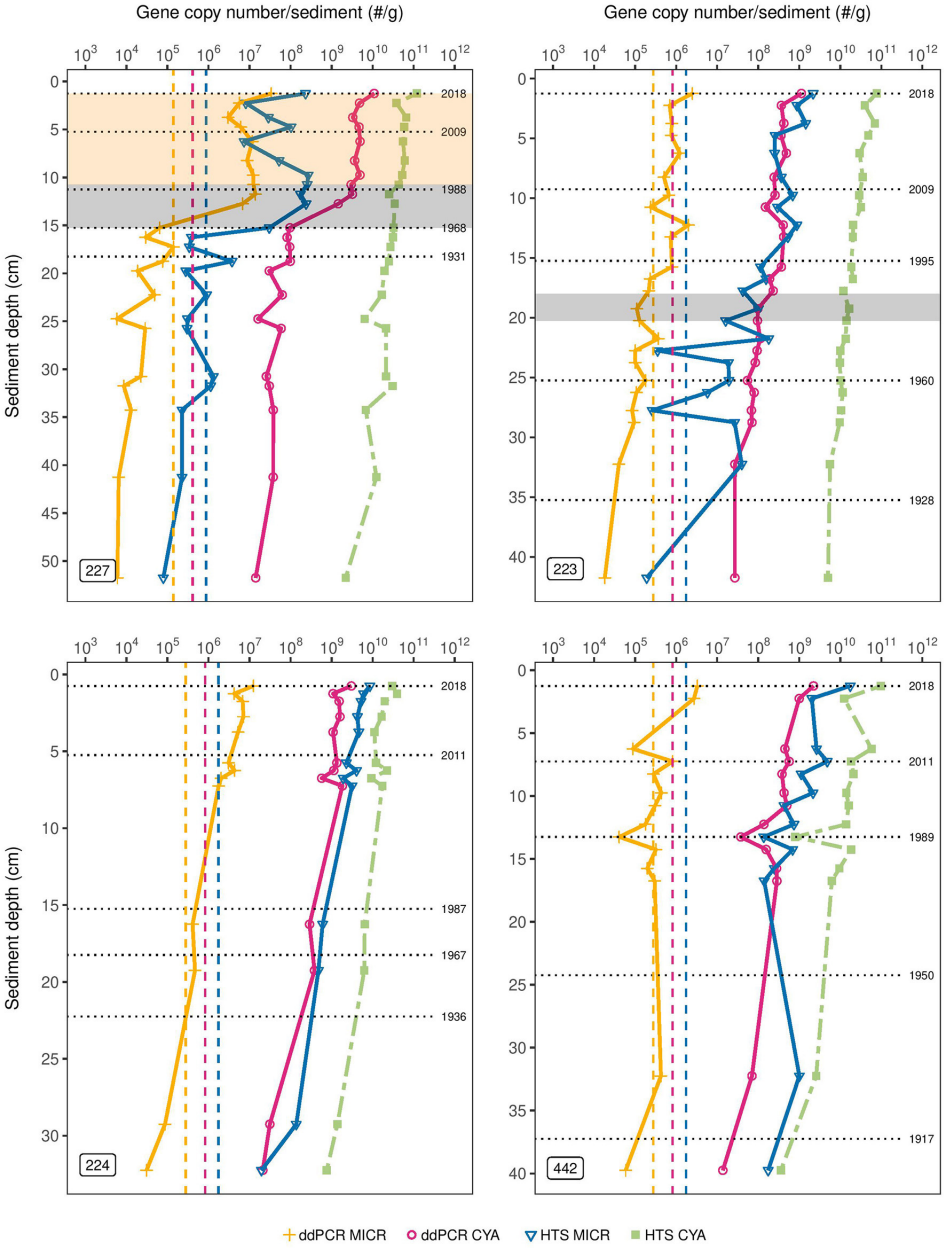
Target gene concentrations in sediment cores of the Experimental Lakes Area, Canada from manipulated lakes 227 and 223 and reference lakes 224 and 442. Absolute gene copy numbers of *Microcystis* 16S rRNA (MICR) and cyanobacterial 16S rRNA (CYA) as determined through droplet digital PCR (ddPCR) are shown, along with the amplicon sequence variant counts of cyanobacterial 16S rRNA and of the *Microcystis* genus from high-throughput sequencing (HTS) (using SILVA). Results are normalized per gram of dry sediment and presented on a logarithmic scale. The orange and pink dashed vertical lines are the detection limits (LODs) for ddPCR-derived *Microcystis* 16s rRNA and cyanobacterial 16S rRNA abundances, respectively. The blue dashed vertical line is the LOD of HTS-derived abundances for both cyanobacterial 16S rRNA and *Microcystis*. The gray shading in the top left plot corresponds to the period of phosphorus and nitrogen loading in Lake 227 (1969–1989), while the beige shading corresponds to the period of phosphorus loading only (1990–2018). The gray shading in the top right plot corresponds to the period of sulfuric acid loading in Lake 223 (1976–1983).

In L227, ddPCR results showed very little change in CYA abundance below 15 cm, with gene copy numbers consistently at ~10^8^ copies/g of dry weight, between two to three orders of magnitude above the corresponding LOD (Figure 1, top left). Subsequently, above 15 cm, total CYA gene copy number increased two orders of magnitude (~100-fold from 10^8^ to 10^10^ copies/g). In contrast, only a gradual ~10-fold increase from 10^10^ to 10^11^ was observed in CYA abundance as determined from HTS throughout the core, and no large increase in abundance was seen around 15 cm. Based on the dating profile from L227 (Supplementary Figure 2), 15.25 cm corresponds to ~1968 (± 2.8 years), the approximate time lake fertilization began. Absolute MICR abundances determined from ddPCR were consistently below the corresponding LOD (1.4 * 10^5^) prior to lake fertilization but subsequently rose approximately two orders of magnitude above detection limits. *Microcystis-*specific 16S rRNA abundances derived from HTS also rose approximately two orders of magnitude above the corresponding LOD for this method (8.8 * 10^5^) at 15 cm. Similar trends were observed when gene copy numbers were normalized per gram of wet sediment (Supplementary Figure 4).

In L223, ddPCR results showed a slight increase in CYA abundance (10-fold from ~10^8^ to 10^9^ copies/g) over time from the bottom of the core to the top (Figure 1, top right). A slight increase in total cyanobacterial abundance was also observed with HTS from ~10^10^ to 10^11^ over time. Experimental acidification of L223 occurred over an 8-year period, between 1976 and 1983, corresponding to ~18–20 cm sediment depth based on the sediment dating profile (Supplementary Figure 2). A small three-fold increase in CYA abundance, determined from ddPCR, was observed during this period, whereas HTS results did not show any increase during this time. The ddPCR-derived MICR concentrations were consistently around the LOD but a three-fold increase above detection limits occurred at ~16 cm. HTS-derived MICR abundances were near or below the corresponding LOD in bottom sediments but rose 100-fold from ~10^7^ to 10^9^ over time.

In L224, a ~10-fold increase in total CYA was observed over time from the bottom of the core to the top in both ddPCR and HTS (Figure 1, bottom left). In L442, a 100-fold increase in CYA was observed over time in both methods (Figure 1, bottom right). *Microcystis-*specific 16S rRNA abundances in L224, as determined from ddPCR, increased 10x above the LOD in top sediments, whereas ddPCR-derived MICR abundances in L442 were consistently at or near the LOD and this remained relatively unchanged over time. *Microcystis-*specific 16S rRNA abundances from HTS in both L224 and L442 increased ~100-fold (10^8^ to 10^10^), but in L442, the profile was skewed toward higher relative abundances in bottom sediments.

**Figure 2:**
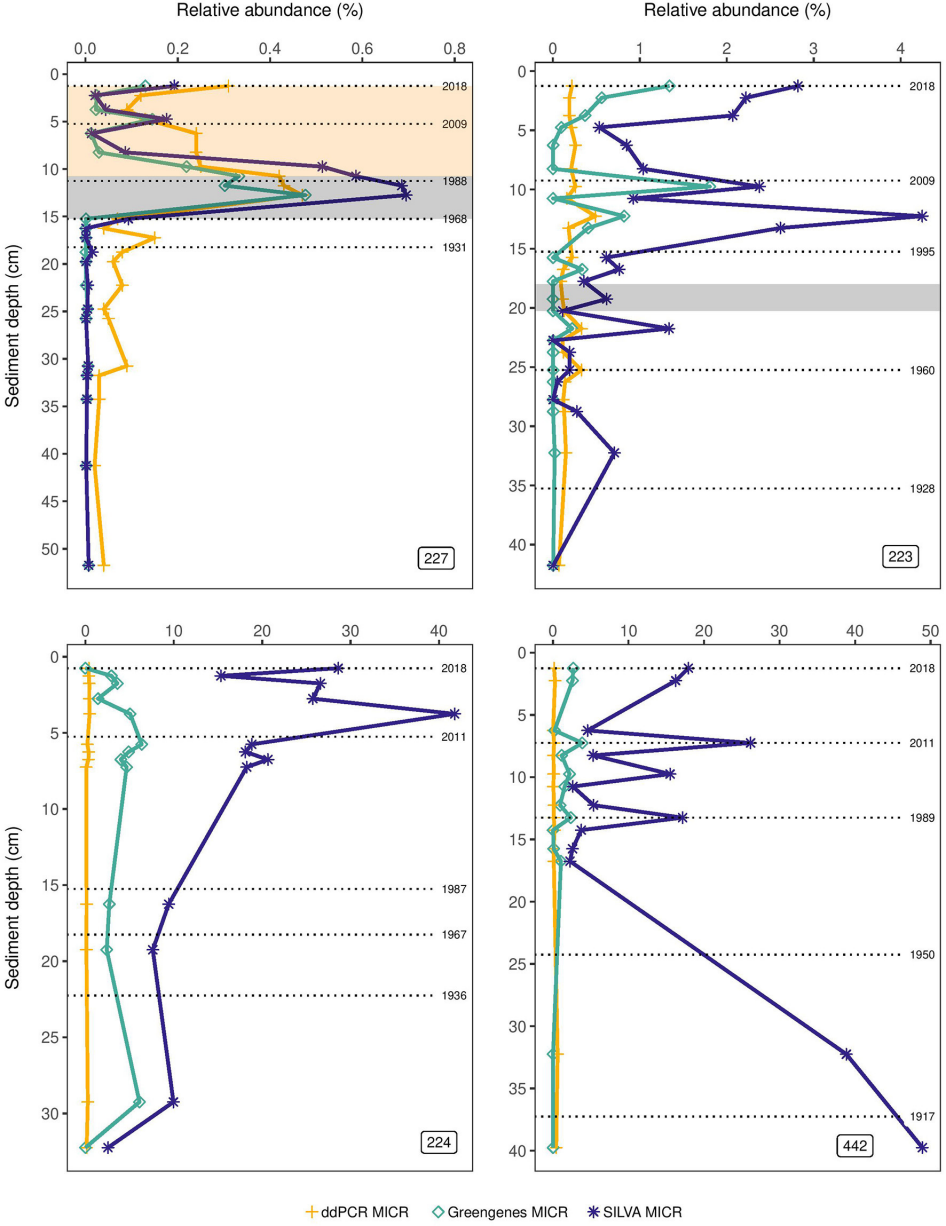
Percent abundance of the *Microcystis* genus (MICR) in lake sediment cores of the Experimental Lakes Area, Canada, from manipulated lakes 227 and 223, along with reference lakes 224 and 442, as determined through droplet digital PCR (ddPCR) and through high-throughput sequencing (HTS) using the SILVA and Greengenes databases. The gray shading in the top left plot corresponds to the period of phosphorus and nitrogen loading in Lake 227 (1969–1989), while the beige shading corresponds to the period of phosphorus loading only (1990–2018). The gray shading in the top right plot corresponds to the period of sulfuric acid loading in Lake 223 (1976–1983).

Relative abundances of MICR derived from ddPCR and from HTS using either the SILVA or Greengenes database showed similar patterns and tracked one another well only in top sediments of L227 (Figure 2). In L227, a rapid increase in MICR was observed with all three approaches at ~15 cm, consistent with the beginning of lake fertilization. All three methods also showed a decline in MICR proportions starting at 11.25 (~1988 ± 2 years—when nitrogen loading stopped). Below 15 cm, the ddPCR-derived MICR proportions appeared to be higher than the HTS results, but the ddPCR absolute abundances were below the LOD in these intervals (see Figure 1). In L223, SILVA and Greengenes appeared to track one another somewhat in top sediments, but relative abundances were much higher than the MICR levels obtained from ddPCR. While ddPCR and Greengenes-derived MICR abundances appeared to not change much throughout the sediment cores of lakes 224 and 442, a large increase over time (5–40%) in L224 was found when using the SILVA database.

**Figure 3:**
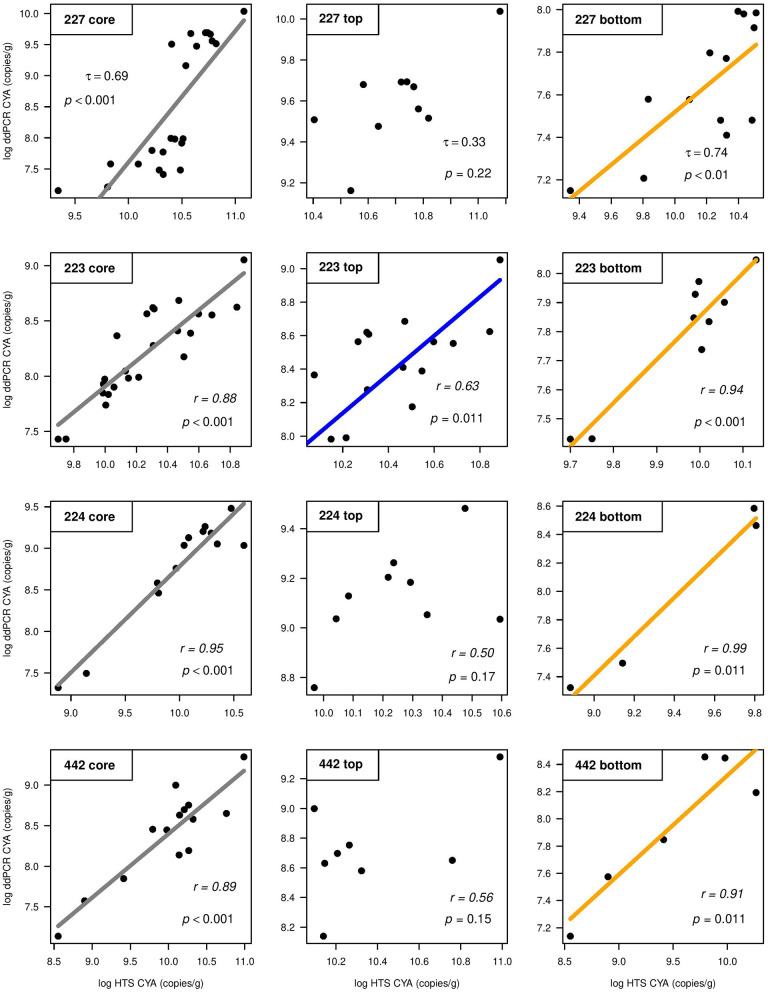
Correlations between log-transformed droplet digital PCR (ddPCR) gene copy numbers and high-throughput sequencing (HTS) amplicon sequence variant counts (using SILVA) for cyanobacterial 16S rRNA (CYA), normalized per gram of dry sediment across the whole core (left), top sediments (middle), and bottom sediments (right) of study lakes 227, 223, 224, and 442. Kendall's τ (Lake 227) and Pearson's *r* (lakes 223, 224, and 442) correlation coefficient values are shown.

Total gene target copy abundance data were normally distributed after log or square root transformation (except for those from L227). Because SILVA and Greengenes were strongly correlated for the number of ASVs assigned to cyanobacteria in all four lakes (Supplementary Figure 5), only the SILVA database was used to compare ddPCR CYA gene copy numbers to HTS-derived CYA counts. In L223, the ACF function showed CYA gene copy numbers derived from ddPCR were temporally autocorrelated, as were the CYA counts from HTS. All correlations were still significant after blockwise permutation. Calculated correlation coefficients showed strong positive relationships between ddPCR and HTS abundance for CYA in all lakes (Figure 3). When cores were split between top and bottom sediments, all four cores showed very strong positive correlations between ddPCR and HTS in bottom sediments (Figure 3, right). While there was a modest correlation in top sediments of L223 (Figure 3, middle), there were no significant correlations between the two methods in top sediments of lakes 227, 224, or 442. Correlation analysis using wet sediments also showed moderate to strong levels of coherence between ddPCR and HTS across the lake sediment cores and in bottom sediments (Supplementary Figure 6).

**Figure 4:**
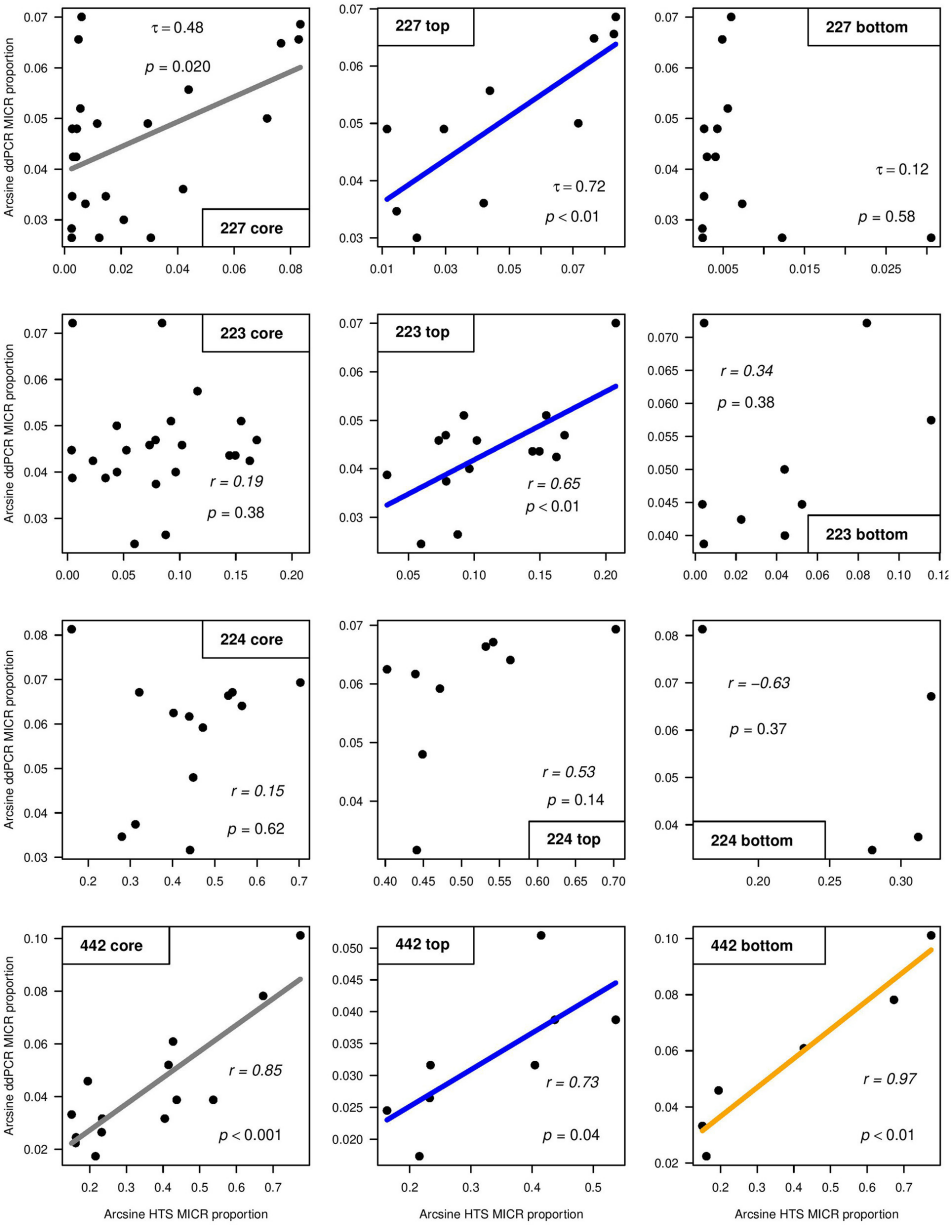
Correlations between arcsine square root transformed droplet digital PCR (ddPCR) and high-throughput sequencing (HTS) (using SILVA) relative abundance outputs for *Microcystis* (MICR) across the whole core (left), top sediments (middle), and bottom sediments (right) of study lakes 227, 223, 224, and 442. Kendall's τ (Lake 227) and Pearson's *r* (lakes 223, 224, and 442) correlation coefficient values are shown.

SILVA and Greengenes taxonomic assignments for the *Microcystis* genus differed (Supplementary Figure 7) and thus both SILVA and Greengenes MICR relative abundances were used to compare with ddPCR-derived MICR relative abundances (Table 3). When using SILVA-based taxonomy, only L227 and L442 showed a significant correlation between ddPCR and HTS for transformed relative MICR abundances. When using the Greengenes database, a correlation between ddPCR- and HTS-derived MICR relative abundances was only found in L227. The SILVA and Greengenes databases were significantly correlated with one another for relative abundances of MICR in lakes 227 and 223. *Microcystis-*specific 16S rRNA abundances derived from ddPCR, SILVA, and Greengenes, were temporally autocorrelated in L227. Correlations were still significant after blockwise permutation. SILVA relative abundance outputs were used to determine the degree of correlation between ddPCR and HTS from top and bottom sediments (Figure 4). Calculated correlation coefficients between top and bottom sediments using Greengenes MICR proportions are presented in Supplementary Figure 8. When using SILVA, moderate to strong correlations were observed in top sediments of lakes 227, 223, and 442, but no significant relationship was found in top sediments of L224. A strong correlation between the two methods was also found in bottom sediments of L442 but not in bottom sediments of the other three lakes.

**Table 3:** Kendall's (L227) and Pearson's (L223, L224, and L442) correlation coefficients of arcsine square root transformed relative *Microcystis* abundances derived from droplet digital PCR (ddPCR) and from high-throughput sequencing (HTS), using the SILVA and Greengenes databases.

			**HTS**	
**Lake**		**ddPCR**	**SILVA**	**Greengenes**
227	ddPCR	1.00	0.48^*^	0.57^**^
	SILVA		1.00	0.72^**^
	Greengenes			1.00
223	ddPCR	1.00	0.19	0.15
	SILVA		1.00	0.81^**^
	Greengenes			1.00
224	ddPCR	1.00	0.15	−0.34
	SILVA		1.00	0.23
	Greengenes			1.00
442	ddPCR	1.00	0.85^**^	−0.31
	SILVA		1.00	−0.02
	Greengenes			1.00

* *p < 0.05*,

** *p < 0.001*.

## Discussion

We compared the sensitivity between ddPCR and HTS by taking advantage of a unique set of experimental and reference lakes and studied the long-term temporal dynamics of cyanobacteria based on the examination of DNA preserved in sediment. Our findings suggest that, while the two methods were significantly correlated with one another, the targeted ddPCR approach reflected more accurately the significant rise in cyanobacteria associated with experimental fertilization.

### Comparison of Absolute Abundances Between ddPCR and HTS at the Level of Cyanobacteria

Our results showed quantification of the cyanobacterial 16S rRNA gene from ddPCR was more consistent with the known history of lake experimentation. In the case of experimental L227, while the ddPCR profile showed a dramatic increase in CYA abundance at 15.25 cm (~1968 ± 2.8 years), HTS did not show any change during lake fertilization. These trends were also observed when gene copy numbers were normalized per gram of wet sediment (Supplementary Figure 4). Eutrophication of L227 began in 1969 and led to the formation of large planktonic cyanobacterial blooms in response to phosphorus and nitrogen loading to the lake (Schindler, 1974, 1977). Phytoplankton records obtained from IISD-ELA show that L227 cyanobacterial biomass increased rapidly in 1969 (Paterson et al., 2011) to over 20 times above that seen in reference lakes at that time. In experimental L223, again only ddPCR illustrated an increase in CYA abundance during the period of experimentation. This is consistent with the surface water phytoplankton records. Findlay and Kasian (1986) found that as pH decreased during the lake acidification experiment, planktonic cyanobacteria increased in biomass. Cyanobacteria mean biomass increased 10-fold from ~0.02 to 0.2 g/m^3^ around 1978, before declining to pre-acidification levels in 1993–1994 (Findlay and Kasian, 1996). The ddPCR results for L223 reflected similar patterns and CYA abundance levels declined to pre-acidification levels at ~11 cm (early 2000s). The HTS-derived results did not demonstrate any increase or decrease during these years.

The lack of an increase in CYA abundance from HTS in L227 and L223 during lake manipulation could be attributed to methodological factors. Primer biases could have impacted the detection and quantification of the cyanobacterial 16S rRNA target gene. The primer sequences used to target CYA in ddPCR have more degenerate bases and amplify a smaller amplicon size than the primers used for HTS. Although both primer sets have been successfully used in other studies (HTS: Nübel et al., 1997; Jungblut et al., 2005; ddPCR: Rinta-Kanto et al., 2005; Pal et al., 2015; Monchamp et al., 2016; Pilon et al., 2019), the choice of specific primers can impact the detection of selected target genes in complex environments (Lee et al., 2012). The amplicon size for the CYA primer set used for HTS was much longer (450 bp) than what was used for ddPCR (269 bp). Cyanobacterial 16S rRNA gene copy number is known to vary between one to four copies per cell among certain cyanobacterial taxa (Schirrmeister et al., 2012). As such, there is likely a greater chance of detecting a taxon that has multiple copies of this 16S rRNA gene if one of those copies has a longer fragment preserved. This may have contributed to the greater overall cyanobacterial abundances observed when using HTS. A study comparing varying PCR amplicon sizes on microbial community analysis found that with shorter amplicons, the PCR reaction proceeded more efficiently and that artifact formation was less likely in comparison to longer fragments (Huber et al., 2009). While the data presented here point to some potential primer bias, it is also likely that the lack of an observable increase in CYA abundance using HTS can be attributed to a high number of false negative errors.

Humic substances have been found to represent potential biases in metabarcoding techniques and other PCR-based approaches on eDNA, generating false-negative results (Rochelle et al., 1992; Savichtcheva et al., 2011; Albers et al., 2013; Thomsen and Willerslev, 2015). Here, most of the samples were taken from the top (recent sediments) such that the impacts of lake manipulation on cyanobacterial dynamics in L223 and L227 could be assessed and compared to the reference lakes which have experienced little to no human impact. While strong correlations were found between the two methods in bottom sediments of both the experimental and reference lakes, no significant relationships between the two estimates of cyanobacterial abundance were observed in top sediments of lakes 227, 224, and 442. Higher levels of humic and fulvic acid substances within surface sediments (~5 cm) (Calace et al., 2006; Hou et al., 2014) may be the reason why top sections of these cores showed no significant relationship between ddPCR and HTS results for CYA gene copy numbers. Humic acids, co-extracted with DNA, can inhibit PCR amplification by suppressing the Taq polymerase, preventing gene targets from amplifying. While the same DNA extractions were used for both ddPCR and HTS, tolerance to inhibitors can differ between the two methods. The ddPCR technology has been found to be highly resistant to PCR inhibitors (Taylor et al., 2017, 2019). DNA templates are randomly partitioned into thousands of droplets of uniform size and volume, improving the performance of the individual endpoint PCR reactions within each droplet (Hindson et al., 2011), reducing the ability of inhibitors to impact amplification, and allowing for reliable detection of low-abundance targets. Low 260:230 ratios from sediment samples have been attributed to high organic contaminants and humic acid levels in other studies (Antony-Babu et al., 2013; Ramírez et al., 2018). The quality of the DNA was in fact particularly low in top sediments of L227; this was expected since the production and deposition of organic matter increased tremendously as a result of the lake fertilization (O'Connell et al., 2020). In this study, only a few samples were selected from the bottom of the reference lake sediment cores because cyanobacterial levels were expected to be low with little variability. Future studies evaluating differences between top and bottom studies (for e.g., studies comparing trends between contemporary and preindustrial sediments) should, however, use larger sample sizes to draw definitive conclusions. In addition to potential humic acid interference, the pooled samples used for sequencing could have also contributed to higher rates of false negatives (Manter et al., 2010).

A major limitation with sedDNA analyses in paleolimnological research includes incomplete DNA extraction or DNA extraction bias. Regardless of adjustments made during the DNA extraction protocol, low DNA yields persisted in a few samples from L227, L223, and L442 sediments, which could have ultimately impacted the number of ASVs passed through the bioinformatic pipeline. A recent study comparing ASV counts across a series of DNA dilutions found that at low DNA concentrations, a disproportionate number of contaminants (from extraction reagents or laboratory environment) were amplified as a result of low signal-to-noise ratio (Caruso et al., 2019). Here, when experimenting with generous truncation parameters within the pipeline (for e.g., using lower quality reads, using only single end reads etc.), the number of ASVs passed through increased overall, but the CYA trends after taxonomy assignment did not differ to the results presented in this study. This indicates that the absolute ASV counts used for analysis were more impacted by the quality and quantity of the starting DNA material, rather than the specific filtering parameters chosen within the pipeline, and that contaminant sequences in samples with low DNA concentrations could have disproportionally been amplified, subsequently reducing the number of ASVs assigned to cyanobacteria. The combined effects of amplified contaminant sequences from some sediment sections and high amounts of false negatives are potential reasons as to the slightly weaker correlations found between ddPCR and HTS when comparing absolute abundances in L227. Based on the results presented here, ddPCR minimizes PCR-inhibiting substances and should be used to quantify absolute concentrations of target genes from sedDNA records.

### Comparison Between ddPCR and HTS Using Relative Abundances of the *Microcystis* Genus

*Microcystis* occurs in these lakes (based on IISD-ELA phytoplankton records), but abundance levels were expected to be low. This allowed for testing the limits of both methods and for low gene copy numbers to be compared between ddPCR and HTS. While strong positive correlations were observed between ddPCR and HTS for absolute cyanobacterial abundances, the combination of overall low *Microcystis* copy numbers derived from ddPCR and little variation in MICR trends, could explain the weaker tracking between the two molecular approaches when using proportion data. The correlation analyses showed moderate to strong levels of agreement between ddPCR and HTS in top sediments of lakes 227, 223, and 442, but results differed based on the use of either the SILVA or Greengenes reference taxonomy database. When using Greengenes, no correlation was observed in top sediments of L223 (Supplementary Figure 8). In addition, the correlations across the core of L442 and across bottom sediments were significant only when using SILVA. This indicates that this more-frequently updated reference database could have picked up more *Microcystis* sequences than Greengenes, which was last updated in 2013. SILVA-derived relative abundances were much higher when compared to Greengenes, and it is possible that misannotations arose, particularly at the genus level in these publicly available DNA reference databases (Sierra et al., 2020). Additionally, SILVA relative *Microcystis* abundances in the reference lakes were ~10–100x higher than in the experimental lakes. Picocyanobacteria (cyanobacteria <2 μm in diameter) and small unicellular coccoid species are abundant in the reference lakes and range from 10^4^ to 10^5^/ml in ELA lakes during the summer (Pick, unpublished data). Both a high level of picocyanobacteria and coccoid species, and annotations to invalid names (in combination with the compositional nature of the sequencing data) could have contributed to the higher SILVA relative MICR abundances within the reference lakes.

Very low absolute MICR abundances and low downcore variation in gene copy numbers (as determined from ddPCR) could have contributed to the insignificant relationship between ddPCR and HTS across the sediment cores of lakes 223 and 224. In addition, the low sample size in L224 could have contributed to the poor statistical power for detecting correlations between the two methods in that core. No firm conclusion can be made regarding the correlation between ddPCR and HTS relative abundances across bottom sediments of the experimental lakes as abundances were found below detection limits in many of those intervals (Figure 1). However, the very high consistency between these two methods in top sediments of L227 likely explains the moderate but significant correlation found across that sediment core. As previously mentioned, it is also possible that contaminants in samples with low DNA concentration reduced the total number of cyanobacterial ASVs in bottom sediments. This could have seriously skewed MICR proportions. Future studies assessing ddPCR and HTS relative abundance data from bottom sediments should use greater sample sizes or interpret results with caution if gene copy numbers are relatively low.

Another potential reason for the discrepancy between ddPCR and HTS for relative MICR abundances could be attributed to the lack of primer specificity. *Microcystis*-specific 16S rRNA primers were used to target the *Microcystis* genus from ddPCR, whereas data filtering through the bioinformatic pipeline was used to obtain *Microcystis* counts from HTS. Both Greengenes and SILVA had relatively higher MICR proportions when compared to ddPCR. It is possible that the MICR primers used in ddPCR were not the most universal and failed to pick up all *Microcystis* present in the samples. While no other *Microcystis* primer sets were tested in this study, future studies should sequence and/or add degenerate bases in the sequence primers to test this hypothesis and confirm the universality of the primers. Rigorous parameters for filtering in the bioinformatics pipeline and the choice of primers selected for analysis should be carefully considered to obtain reliable proportional data for the accurate inference of the composition.

### Recommendations

The method of choice for sedDNA research should depend on the ultimate goals of the study. Here, we found ddPCR to have fewer limitations than HTS, and ddPCR results were more consistent with the history of cyanobacteria in these lakes. The high-throughput capacity of ddPCR and its ability to tolerate inhibitors makes this molecular method more suitable for quantifying targets from complex environments like sediments. Absolute abundance data from HTS, abundance measures derived from top sediments, or samples with very low gene copy numbers, should be interpreted with caution. However, metabarcoding approaches provide unparalleled resolution of microbial community diversity and expand taxonomic scope at unprecedented scales. If the main goal of a study is to track abundance through time in an environment with a *priori* knowledge that low levels of inhibitors are present in the samples (based on nanodrop quality scores, for example), then either ddPCR or HTS are appropriate methods to consider. However, if the complexity of the environment is unknown, or if high levels of PCR inhibitors such as organic contaminants or humic acids are likely to be found, ddPCR is likely the more appropriate choice. If only relative abundance counts are necessary, then HTS alone is sufficient (although the choice of database for taxonomic assignment should be carefully considered). On the other hand, if overall community dynamics are assessed, and both abundance and community composition are required, we recommend to first analyze results through HTS and subsequently quantify change through time with ddPCR. As both ddPCR and HTS methods continue to improve and as costs continue to decline, the benefits of using both approaches in tandem can increase the validity of using sedDNA to reconstruct historical dynamics.

## Data Availability Statement

The datasets presented in this study can be found in online repositories. The name of the repository and accession number can be found below: National Center for Biotechnology Information (NCBI) BioProject, https://www.ncbi.nlm.nih.gov/bioproject/, PRJNA679845.

## Author Contributions

HM and FP designed the study. HM and AB sectioned the sediment cores. IG-E provided sterile space and assistance with sediment-sample preparation. AB performed the varve counting. HM performed the molecular experiments, analyzed the data, and wrote the first draft of the manuscript. WD, AB, IG-E, and FP revised the manuscript. All authors contributed to the article and approved the submitted version.

## Conflict of Interest

The authors declare that the research was conducted in the absence of any commercial or financial relationships that could be construed as a potential conflict of interest.

## References

[B1] Albers C. N. Jensen A. Bælum J. Jacobsen C. S. (2013). Inhibition of DNA polymerases used in Q-PCR by structurally different soil-derived humic substances. Geomicrobiol. J. 30, 675–681. 10.1080/01490451.2012.758193

[B2] Anderson R. F. Schiff S. L. Hesslein R. H. (1987). Determining sediment accumulation and mixing rates using ^210^Pb, ^137^Cs and other tracers: problems due to post-depositional mobility or coring artifacts. Can. J. Fish. Aquat. Sci. 44, 231–250. 10.1139/f87-29811381746

[B3] Antony-Babu S. Murat C. Deveau A. Le Tacon F. Frey-Klett P. Uroz S. (2013). An improved method compatible with metagenomic analyses to extract genomic DNA from soils in *Tuber melanosporum* orchards. J. Appl. Microbiol. 115, 163–170. 10.1111/jam.12205 23581622

[B4] Armbruster D. A. Pry T. (2008). Limit of blank, limit of detection and limit of quantitation. Clin. Biochem. Rev. 29(Suppl 1), S49–S52. 18852857 PMC2556583

[B5] Blais J. M. Kalff J. Cornett J. R. Evans D. R. (1995). Evaluation of ^210^Pb dating in lake sediments using stable Pb, *Ambrosia* pollen, and ^137^Cs. J. Paleolimnol. 13, 169–178. 10.1007/BF00678105

[B6] Bolyen E. Rideout J. R. Dillon M. R. Bokulich N. A. Abnet C. C. Al-Ghalith G. A.. (2019). Reproducible, interactive, scalable and extensible microbiome data science using QIIME 2. Nat. Biotechnol. 37, 852–857. 10.1038/s41587-019-0209-9 31341288 PMC7015180

[B7] Brazeau M. L. Blais J. M. Paterson A. M. Keller W. Poulain A. J. (2013). Evidence for microbially mediated production of elemental mercury (Hg^0^) in subarctic lake sediments. Appl. Geochemistry 37, 142–148. 10.1016/j.apgeochem.2013.07.020

[B8] Bylemans J. Gleeson D. M. Duncan R. P. Hardy C. M. Furlan E. M. (2019). A performance evaluation of targeted eDNA and eDNA metabarcoding analyses for freshwater fishes. eDNA 1, 402–414. 10.1002/edn3.41

[B9] Calace N. Cardellicchio N. Petronio B. M. Pietrantonio M. Pietroletti M. (2006). Sedimentary humic substances in the northern Adriatic sea (Mediterranean sea). Mar. Environ. Res. 61, 40–58. 10.1016/j.marenvres.2005.05.002 16019060

[B10] Caruso V. Song X. Asquith M. Karstens L. (2019). Performance of microbiome sequence inference methods in environments with varying biomass. mSystems 4:e00163–18. 10.1128/mSystems.00163-18 30801029 PMC6381225

[B11] Coolen M. J. L. Boere A. Abbas B. Baas M. Wakeham S. G. Sinninghe Damsté J. S. (2006). Ancient DNA derived from alkenone-biosynthesizing haptophytes and other algae in Holocene sediments from the Black Sea. Paleoceanography 21:PA1005. 10.1029/2005PA001188

[B12] Cruikshank D. R. (1984). Whole *Lake Chemical Additions* in the Experimental Lakes Area, 1969−*1983*. Canadian Data Report of Fisheries and Aquatic Sciences No. 449.

[B13] Crusius J. Anderson R. F. (1995). Evaluating the mobility of ^137^Cs, ^239+240^Pu and ^210^Pb from their distribution in laminated lake sediments. J. Paleolimnol. 13, 119–141. 10.1007/BF00678102

[B14] Domaizon I. Winegardner A. Capo E. Gauthier J. Gregory-Eaves I. (2017). DNA-based methods in paleolimnology: new opportunities for investigating long-term dynamics of lacustrine biodiversity. J. Paleolimnol. 58, 1–21. 10.1007/s10933-017-9958-y

[B15] Emerson S. Hesslein R. (1973). Distribution and uptake of artificially introduced radium-226 in a small lake. J. Fish. Res. Board Canada 30, 1485–1490. 10.1139/f73-238

[B16] Findlay D. L. Kasian S. E. M. (1986). Phytoplankton community responses to acidification of lake 223, experimental lakes area, northwestern Ontario. Water Air Soil Pollut. 30, 719–726. 10.1007/BF00303337

[B17] Findlay D. L. Kasian S. E. M. (1990). Phytoplankton communities of lakes experimentally acidified with sulfuric and nitric acids. Can. J. Fish. Aquat. Sci. 47, 1378–1386. 10.1139/f90-157

[B18] Findlay D. L. Kasian S. E. M. (1996). The effect of incremental pH recovery on the Lake 223 phytoplankton community. Can. J. Fish. Aquat. Sci. 53, 856–864. 10.1139/cjfas-53-4-856

[B19] Glass D. C. Gray C. N. (2001). Estimating mean exposure from censored data: exposure to benzene in the Australian petroleum industry. Ann. Occup. Hyg. 45, 275–282. 10.1016/S0003-4878(01)00022-9 11378148

[B20] Hamaguchi M. Shimabukuro H. Hori M. Yoshida G. Terada T. Miyajima T. (2018). Quantitative real-time polymerase chain reaction (PCR) and droplet digital PCR duplex assays for detecting *Zostera marina* DNA in coastal sediments. Limnol. Oceanogr. Methods 16, 253–264. 10.1002/lom3.10242

[B21] Hänfling B. Handley L. L. Read D. S. Hahn C. Li J. Nichols P.. (2016). Environmental DNA metabarcoding of lake fish communities reflects long-term data from established survey methods. Mol. Ecol. 25, 3101–3119. 10.1111/mec.13660 27095076

[B22] Harke M. J. Steffen M. M. Gobler C. J. Otten T. G. Wilhelm S. W. Wood S. A.. (2016). A review of the global ecology, genomics, and biogeography of the toxic cyanobacterium, *Microcystis* spp. Harmful Algae 54, 4–20. 10.1016/j.hal.2015.12.007 28073480

[B23] Harper L. R. Handley L. L. Hahn C. Boonham N. Rees H. S. Gough K. C.. (2018). Needle in a haystack? A comparison of eDNA metabarcoding and targeted qPCR for detection of the great crested newt (Triturus cristatus). Ecol. Evol. 8, 6330–6341. 10.1002/ece3.4013 29988445 PMC6024127

[B24] Hesslein R. H. Broecker W. S. Quay P. D. Schindler D. W. (1980b). Whole-lake radioarbon experiment in an oligotrophic lake at the Experimental Lakes Area, northwestern Ontario. Can. J. Fish. Aquat. Sci. 37, 454–463. 10.1139/f80-059

[B25] Hesslein R. H. Broecker W. S. Schindler D. W. (1980a). Fates of metal radiotracers added to a whole lake: sediment-water interactions. Can. J. Fish. Aquat. Sci. 37, 378–386. 10.1139/f80-052

[B26] Higgins S. N. Paterson M. J. Hecky R. E. Schindler D. W. Venkiteswaran J. J. Findlay D. L. (2018). Biological nitrogen fixation prevents the response of a eutrophic lake to reduced loading of nitrogen: evidence from a 46-year whole-lake experiment. Ecosystems 21, 1088–1100. 10.1007/s10021-017-0204-2

[B27] Hindson B. J. Ness K. D. Masquelier D. A. Belgrader P. Heredia N. J. Makarewicz A. J.. (2011). High-throughput droplet digital PCR system for absolute quantitation of DNA copy number. Anal. Chem. 83, 8604–8610. 10.1021/ac202028g 22035192 PMC3216358

[B28] Hong S. Bunge J. Leslin C. Jeon S. Epstein S. S. (2009). Polymerase chain reaction primers miss half of rRNA microbial diversity. ISME J. 3, 1365–1373. 10.1038/ismej.2009.89 19693101

[B29] Hou D. He J. Lü C. Wang W. Zhang F. (2014). Spatial distributions of humic substances and evaluation of sediment organic index on Lake Dalinouer, China. J. Geochem. 2014, 1–13. 10.1155/2014/502597

[B30] Huber J. A. Morrison H. G. Huse S. M. Neal P. R. Sogin M. L. Welch D. B. M. (2009). Effect of PCR amplicon size on assessments of clone library microbial diversity and community structure. Environ. Microbiol. 11, 1292–1302. 10.1111/j.1462-2920.2008.01857.x 19220394 PMC2716130

[B31] Jungblut A. Hawes I. Mountfort D. Hitzfeld B. Dietrich D. Burns B.. (2005). Diversity within cyanobacterial mat communities in variable salinity meltwater ponds of McMurdo Ice Shelf, Antarctica. Environ. Microbiol. 7, 519–529. 10.1111/j.1462-2920.2005.00717.x 15816929

[B32] Lamb P. D. Hunter E. Pinnegar J. K. Creer S. Davies R. G. Taylor M. I. (2018). How quantitative is metabarcoding: a meta-analytical approach. Mol. Ecol. 28, 420–430. 10.1111/mec.14920 30408260 PMC7379500

[B33] Lamothe K. A. Jackson D. A. Somers K. M. (2018). Long-term directional trajectories among lake crustacean zooplankton communities and water chemistry. Can. J. Fish. Aquat. Sci. 75, 1926–1939. 10.1139/cjfas-2017-0518

[B34] Leavitt P. R. Findlay D. L. (1994). Comparison of fossil pigments with 20 years of phytoplankton data from eutrophic Lake 227, Experimental Lakes Area, Ontario. Can. J. Fish. Aquat. Sci. 51, 2286–2299. 10.1139/f94-232

[B35] Lee C. K. Herbold C. W. Polson S. W. Wommack K. E. Williamson S. J. McDonald I. R.. (2012). Groundtruthing next-gen sequencing for microbial ecology-biases and errors in community structure estimates from PCR amplicon pyrosequencing. PLoS ONE 7:44224. 10.1371/journal.pone.0044224 22970184 PMC3435322

[B36] Legendre P. (1993). Spatial autocorrelation: trouble or new paradigm? Ecology 74, 1659–1673. 10.2307/1939924

[B37] Legendre P. Legendre L. (2012). Numerical Ecology, 3rd Edn. Amsterdam: Elsevier.

[B38] Legrand B. Lamarque A. Sabart M. Latour D. (2017). Benthic archives reveal recurrence and dominance of toxigenic cyanobacteria in a eutrophic lake over the last 220 years. Toxins 9:271. 10.3390/toxins9090271 28869578 PMC5618204

[B39] Manter D. K. Weir T. L. Vivanco J. M. (2010). Negative effects of sample poooling on PCR-based estimates of soil microbial richness and community strucutre. Appl. Environ. Microbiol. 76, 2086–2090. 10.1128/AEM.03017-09 20139317 PMC2849261

[B40] Monchamp M.-E. Spaak P. Domaizon I. Dubois N. Bouffard D. Pomati F. (2018). Homogenization of lake cyanobacterial communities over a century of climate change and eutrophication. Nat. Ecol. Evol. 2, 317–324. 10.1038/s41559-017-0407-0 29230026

[B41] Monchamp M.-E. Walser J.-C. Pomati F. Spaak P. (2016). Sedimentary DNA reveals cyanobacterial community diversity over 200 years in two peri-alpine lakes. Appl. Environ. Microbiol. 82, 6472–6482. 10.1128/AEM.02174-16 27565621 PMC5066364

[B42] Nathan L. M. Simmons M. Wegleitner B. J. Jerde C. L. Mahon A. R. (2014). Quantifying environmental DNA signals for aquatic invasive species across multiple detection platforms. Environ. Sci. Technol. 48, 12800–12806. 10.1021/es5034052 25299381

[B43] Nübel U. Garcia-Pichel F. Muyzer G. (1997). PCR primers to amplify 16S rRNA genes from cyanobacteria. Appl. Environ. Microbiol. 63, 3327–3332. 10.1128/aem.63.8.3327-3332.1997 9251225 PMC168636

[B44] O'Connell D. W. Ansems N. Kukkadapu R. K. Jaisi D. Orihel D. M. Cade-Menun B. J.. (2020). Changes in sedimentary phosphorus burial following artificial eutrophication of Lake 227, Experimental Lakes Area, Ontario, Canada. J. Geophys. Res. 125:e2020JG005713. 10.1029/2020jg005713

[B45] Pal S. Gregory-Eaves I. Pick F. R. (2015). Temporal trends in cyanobacteria revealed through DNA and pigment analyses of temperate lake sediment cores. J. Paleolimnol. 54, 87–101. 10.1007/s10933-015-9839-1

[B46] Parducci L. Bennett K. D. Ficetola G. F. Alsos I. G. Suyama Y. Wood J. R.. (2017). Ancient plant DNA in lake sediments. New Phytol. 214, 924–942. 10.1111/nph.14470 28370025

[B47] Paterson M. J. Schindler D. W. Hecky R. E. Findlay D. L. Rondeau K. J. (2011). Comment: Lake 227 shows clearly that controlling inputs of nitrogen will not reduce or prevent eutrophication of lakes. Limnol. Oceanogr. 56, 1545–1547. 10.4319/lo.2011.56.4.1545

[B48] Pilon S. Zastepa A. Taranu Z. E. Gregory-Eaves I. Racine M. Blais J. M.. (2019). Contrasting histories of microcystin-producing cyanobacteria in two temperate lakes as inferred from quantitative sediment DNA analyses. Lake Reserv. Manag. 35, 102–117. 10.1080/10402381.2018.1549625

[B49] Poulain A. J. Aris-Brosou S. Blais J. M. Brazeau M. Keller W. Paterson A. M. (2015). Microbial DNA records historical delivery of anthropogenic mercury. ISME J. 9, 2541–2550. 10.1038/ismej.2015.86 26057844 PMC4817628

[B50] Quay P. D. Broecker W. S. Hesslein R. H. Schindler D. W. (1980). Vertical diffusion rates determined by tritium tracer experiments in the thermocline and hypolimnion of two lakes. Limnol. Oceanogr. 25, 201–218. 10.4319/lo.1980.25.2.0201

[B51] Ramírez G. A. Graham D. D'Hondt S. (2018). Influence of commercial DNA extraction kit choice on prokaryotic community metrics in marine sediment. Limnol. Oceanogr. Methods. 16, 525–536. 10.1002/lom3.10264

[B52] Rinta-Kanto J. M. Ouellette A. J. A. Boyer G. L. Twiss M. R. Bridgeman T. B. Wilhelm S. W. (2005). Quantification of toxic *Microcystis* spp. during the 2003 and 2004 blooms in western Lake Erie using quantitative real-time PCR. Environ. Sci. Technol. 39, 4198–4205. 10.1021/es048249u 15984800

[B53] Rochelle P. A. Fry J. C. John Parkes R. Weightman A. J. (1992). DNA extraction for 16S rRNA gene analysis to determine genetic diversity in deep sediment communities. FEMS Microbiol. Lett. 100, 59–65. 10.1111/j.1574-6968.1992.tb14019.x 1282488

[B54] Savichtcheva O. Debroas D. Kurmayer R. Villar C. Jenny J. P. Arnaud F.. (2011). Quantitative PCR enumeration of total/toxic *Planktothrix rubescens* and total cyanobacteria in preserved DNA isolated from lake sediments. Appl. Environ. Microbiol. 77, 8744–8753. 10.1128/AEM.06106-11 21984244 PMC3233095

[B55] Schindler D. W. (1974). Eutrophication and recovery in experimental lakes: implications for lake management. Science 184, 897–899. 10.1126/science.184.4139.897 17782381

[B56] Schindler D. W. (1977). Evolution of phosphorus limitation in lakes. Science 195, 260–262. 10.1126/science.195.4275.260 17787798

[B57] Schindler D. W. Bayley S. E. Parker B. R. Beaty K. G. Cruikshank D. R. Fee E. J.. (1996). The effects of climatic warming on the properties of boreal lakes and streams at the Experimental Lakes Area, northwestern Ontario. Limnol. Oceanogr. 41, 1004–1017. 10.4319/lo.1996.41.5.1004

[B58] Schindler D. W. Beaty K. G. Fee E. J. Cruikshank D. R. DeBruyn E. R. Findlay D. L.. (1990). Effects of climatic warming on lakes of the central boreal forest. Science 250, 967–970. 10.1126/science.250.4983.967 17746921

[B59] Schindler D. W. Hecky R. E. Findlay D. L. Stainton M. P. Parker B. R. Paterson M. J.. (2008). Eutrophication of lakes cannot be controlled by reducing nitrogen input: results of a 37-year whole-ecosystem experiment. Proc. Natl. Acad. Sci. U.S.A. 105, 11254–11258. 10.1073/pnas.0805108105 18667696 PMC2491484

[B60] Schindler D. W. Wagemann R. Cook R. B. Ruszczynski T. Prokopowich J. (1980). Experimental acidification of Lake 223, Experimental Lakes Area: background data and the first three years of acidification. Can. J. Fish. Aquat. Sci. 37, 342–354. 10.1139/f80-048

[B61] Schirrmeister B. E. Dalquen D. A. Anisimova M. Bagheri H. C. (2012). Gene copy number variation and its significance in cyanobacterial phylogeny. BMC Microbiol. 12:177. 10.1186/1471-2180-12-177 22894826 PMC3552681

[B62] Shelton A. O. O'Donnell J. L. Samhouri J. F. Lowell N. Williams G. D. Kelly R. P. (2016). A framework for inferring biological communities from environmental DNA. Ecol. Appl. 26, 1645–1659. 10.1890/15-1733.1 27755698

[B63] Sierra M. A. Li Q. Pushalkar S. Paul B. Sandoval T. A. Kamer A. R.. (2020). The influences of bioinformatics tools and reference databases in analyzing the human oral microbial community. Genes 11:878. 10.3390/genes11080878 32756341 PMC7465726

[B64] Singh G. Sithebe A. Enitan A. M. Kumari S. Bux F. Stenström T. A. (2017). Comparison of droplet digital PCR and quantitative PCR for the detection of *Salmonella* and its application for river sediments. J. Water Health 15, 505–508. 10.2166/wh.2017.259 28771147

[B65] Smol J. P. (2008). Pollution of Lakes and Rivers: A Paleoenvironmental Perspective, 2nd Edn. Malden, MA: Wiley-Blackwell Publishing.

[B66] Taberlet P. Bonin A. Zinger L. Coissac E. (2018). Evironmental DNA: For Biodiversity Research and Monitoring. Oxford; New York, NY: Oxford University Press.

[B67] Taranu Z. E. Carpenter S. R. Frossard V. Jenny J.-P. Thomas Z. Vermaire J. C.. (2018). Can we detect ecosystem critical transitions and signals of changing resilience from paleo-ecological records? Ecosphere 9:e02438. 10.1002/ecs2.2438

[B68] Taylor S. C. Laperriere G. Germain H. (2017). Droplet digital PCR versus qPCR for gene expression analysis with low abundant targets: from variable nonsense to publication quality data. Sci. Rep. 7:2409. 10.1038/s41598-017-02217-x 28546538 PMC5445070

[B69] Taylor S. C. Nadeau K. Abbasi M. Lachance C. Nguyen M. Fenrich J. (2019). The ultimate qPCR experiment: producing publication quality, reproducible data the first time. Trends Biotechnol. 37, 761–774. 10.1016/j.tibtech.2018.12.002 30654913

[B70] Thomsen P. F. Willerslev E. (2015). Environmental DNA - an emerging tool in conservation for monitoring past and present biodiversity. Biol. Conserv. 183, 4–18. 10.1016/j.biocon.2014.11.019

[B71] Tse T. J. Doig L. E. Tang S. Zhang X. Sun W. Wiseman S. B.. (2018). Combining high-throughput sequencing of *seda*DNA and traditional paleolimnological techniques to infer historical trends in cyanobacterial communities. Environ. Sci. Technol. 52, 6842–6853. 10.1021/acs.est.7b06386 29782156

[B72] United States Environmental Protection Agency (2006). Data Quality Assessment: Statistical Methods for Practitioners. Washington, DC: Office of Environmental Information.

[B73] Urbach E. Robertson D. L. Chisholm S. W. (1992). Multiple evolutionary origins of prochlorophytes within the cyanobacterial radiation. Nature 355, 267–270. 10.1038/355267a0 1731225

[B74] Ushio M. Murakami H. Masuda R. Sado T. Miya M. Sakurai S.. (2018). Quantitative monitoring of multispecies fish environmental DNA using high-throughput sequencing. MBMG 2:e23297. 10.1101/113472

[B75] Verschuren D. (2000). Freeze coring soft sediments in tropical lakes. J. Paleolimnol. 24, 361–365. 10.1023/A:1008191418497

[B76] Wood S. A. Pochon X. Laroche O. von Ammon U. Adamson J. Zaiko A. (2019). A comparison of droplet digital polymerase chain reaction (PCR), quantitative PCR and metabarcoding for species-specific detection in environmental DNA. Mol. Ecol. Resour. 19, 1407–1419. 10.1111/1755-0998.13055 31293089

[B77] Yeates C. Gillings M. R. Davison A. D. Altavilla N. Veal D. A. (1997). PCR amplification of crude microbial DNA extracted from soil. Lett. Appl. Microbiol. 25, 303–307. 10.1046/j.1472-765x.1997.00232.x 9351282

[B78] Zhou J. Bruns M. A. Tiedje J. M. (1996). DNA recovery from soils of diverse composition. Appl. Environ. Microbiol. 62, 316–322. 10.1128/AEM.62.2.316-322.1996 8593035 PMC167800

