# Supplementary material for: Comparing Quantitative Methods for Analyzing Sediment DNA Records of Cyanobacteria in Experimental and Reference Lakes
Source: Front Microbiol. 2021 Jun 18;12:669910. doi: 10.3389/fmicb.2021.669910 (PMC8250803; doi:10.3389/fmicb.2021.669910)
Supplement: Supplementary file 1 [file Image_1.PDF]

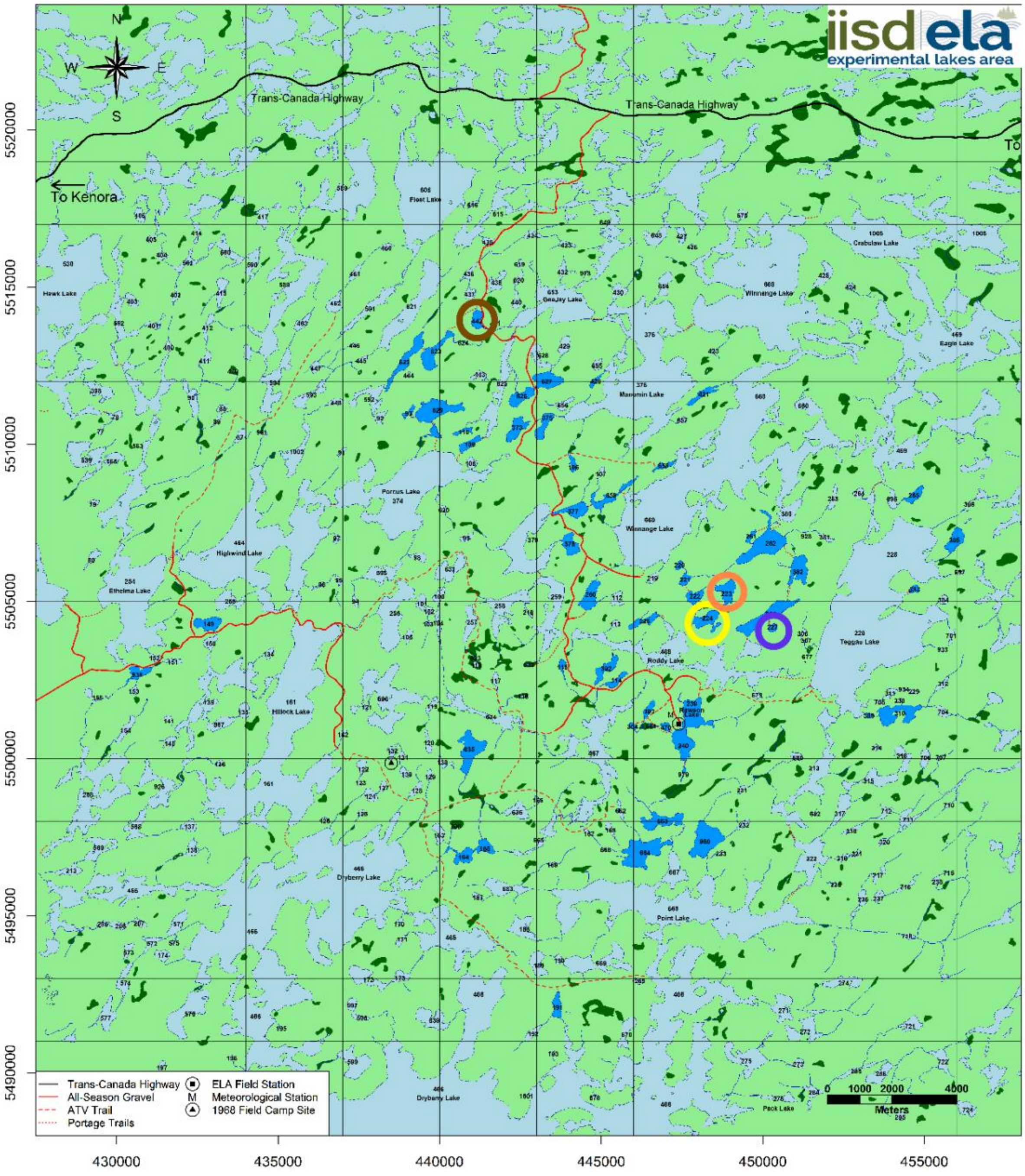

Figure S1. Map of the IISD Experimental Lakes Area. Circled sites correspond to study lakes 227 (purple), 223 (orange), 224 (yellow), and 442 (brown). Map from IISD Experimental Lakes Area (iisd.org/ela).
