# Supplementary figures and images for: Comparing Quantitative Methods for Analyzing Sediment DNA Records of Cyanobacteria in Experimental and Reference Lakes
Source: Front Microbiol. 2021 Jun 18;12:669910. doi: 10.3389/fmicb.2021.669910 (PMC8250803; doi:10.3389/fmicb.2021.669910)

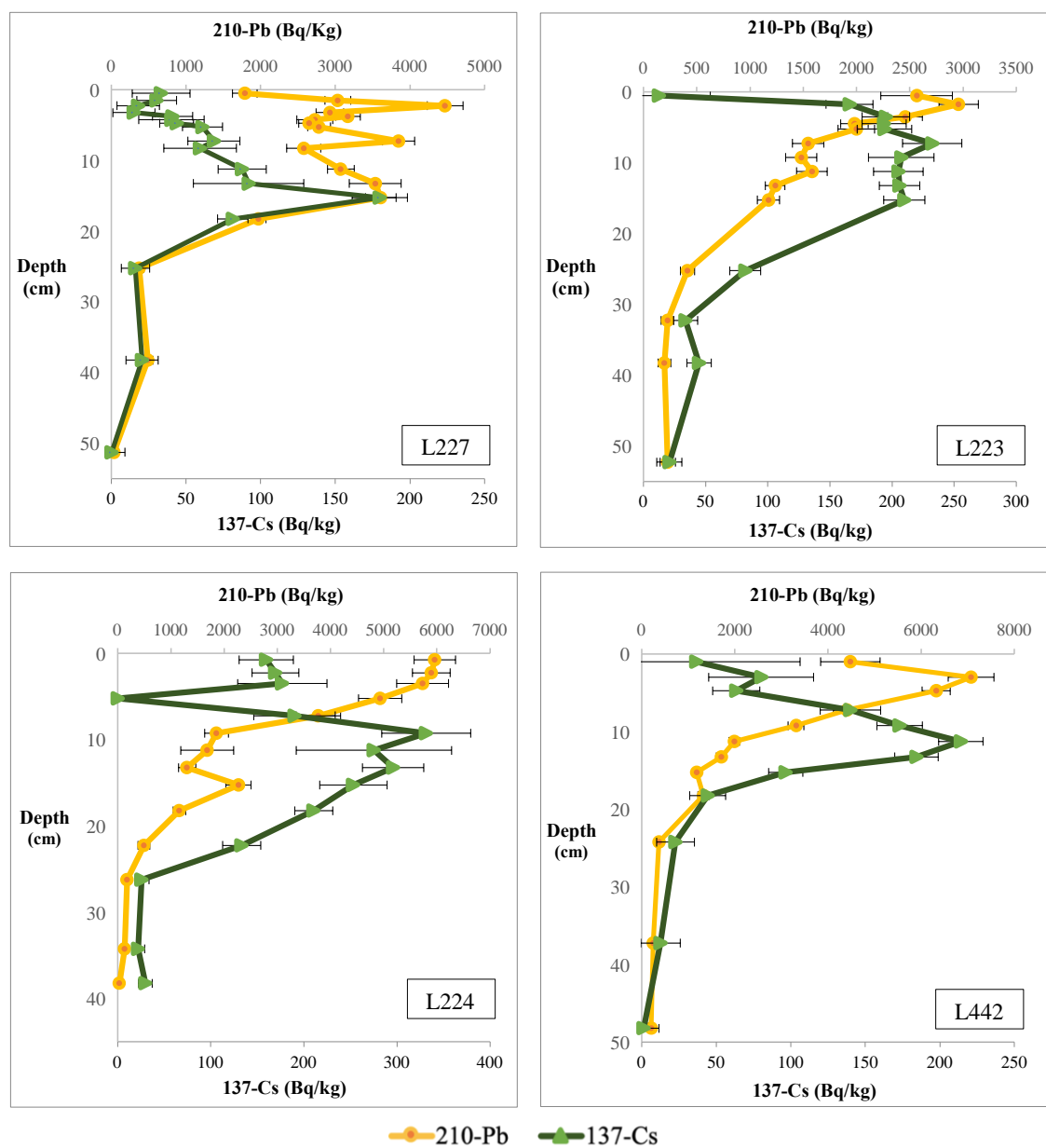

Figure S2. Age-depth models of sediments from ELA lakes 227, 223, 224, and 442 showing  $^{210}\text{Pb}$  and  $^{137}\text{Cs}$  profiles.

Supplement: Supplementary file 2 [file Image_2.PDF]

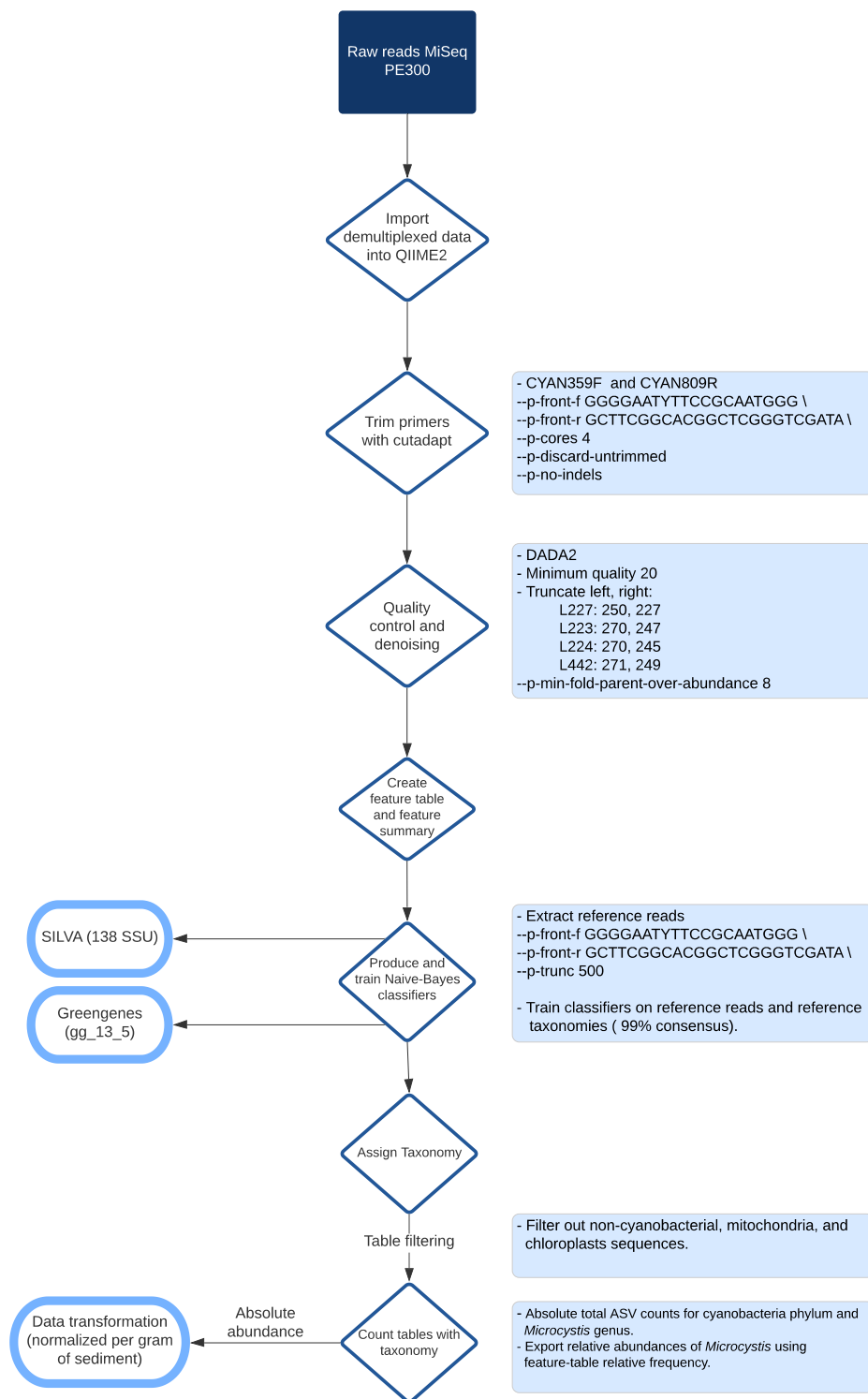

Figure S3. Summary of the main steps applied for the analysis of the sequencing data.

Supplement: Supplementary file 3 [file Image_3.PDF]

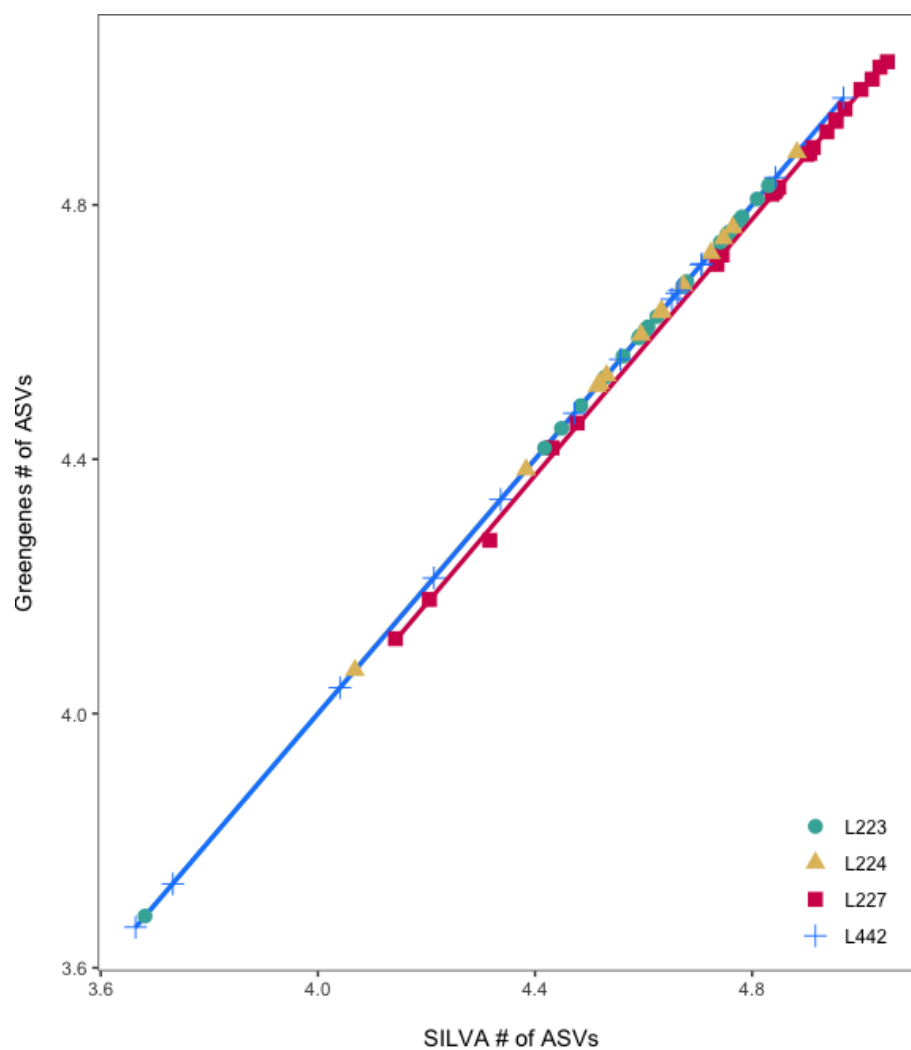

Supplement: Supplementary file 5 [file Image_5.PDF]
