# Supplementary material for: Comparing Quantitative Methods for Analyzing Sediment DNA Records of Cyanobacteria in Experimental and Reference Lakes
Source: Front Microbiol. 2021 Jun 18;12:669910. doi: 10.3389/fmicb.2021.669910 (PMC8250803; doi:10.3389/fmicb.2021.669910)
Supplement: Supplementary file 4 [file Image_4.PDF]

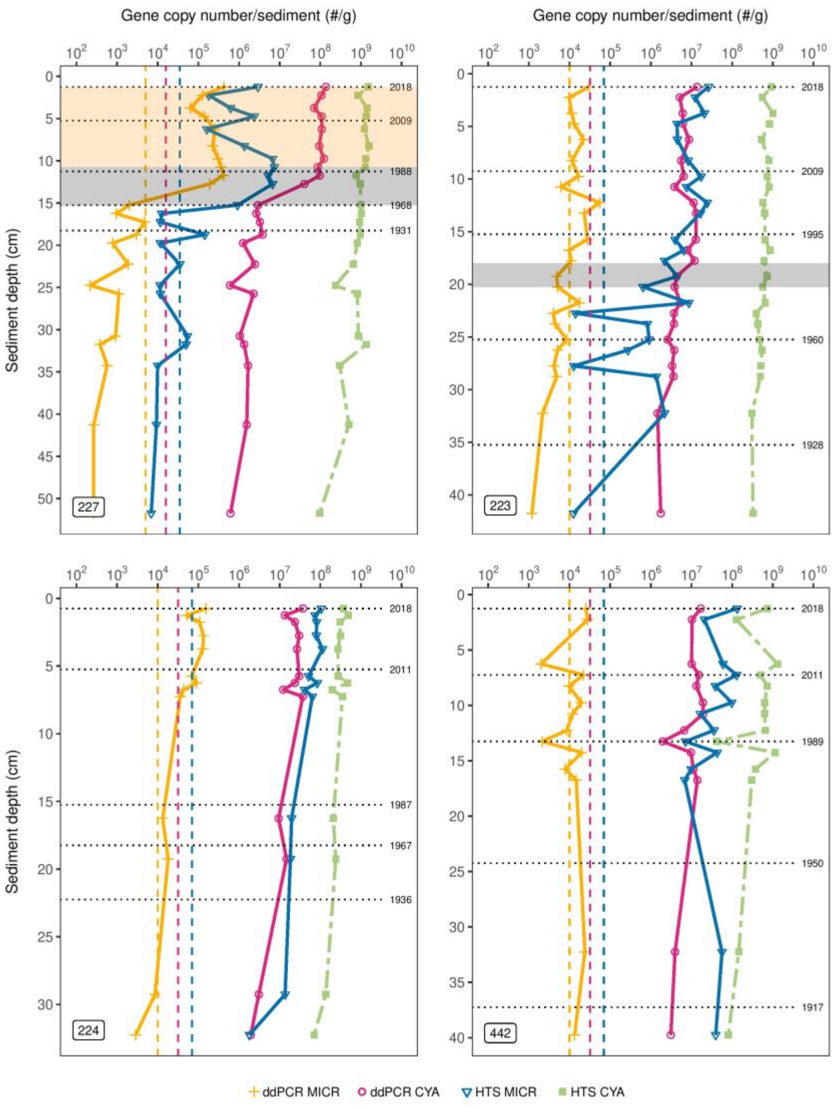

Figure S4. Target gene concentrations in sediment cores of the Experimental Lakes Area, Canada from manipulated lakes 227 and 223 and reference lakes 224 and 442. Absolute gene copy numbers of *Microcystis* 16S rRNA (MICR) and cyanobacterial 16S rRNA (CYA) as determined through droplet digital PCR (ddPCR) are shown, along with the amplicon sequence variant counts of cyanobacterial 16S rRNA and of the *Microcystis* genus from high-throughput sequencing (HTS) (using SILVA). Results are normalized per gram of wet sediment and presented on a logarithmic scale. The orange and pink dashed vertical lines are the detection limits (LODs) for ddPCR-derived *Microcystis* 16s rRNA and cyanobacterial 16S rRNA abundances, respectively. The blue dashed vertical line is the LOD of HTS-derived abundances for both cyanobacterial 16S rRNA and *Microcystis*. The grey shading in the top left plot corresponds to the period of phosphorus and nitrogen loading in Lake 227 (1969-1989), while the beige shading corresponds to the period of phosphorus loading only (1990-2018). The grey shading in the top right plot corresponds to the period of sulfuric acid loading in Lake 223 (1976-1983).
