# Supplementary material for: Comparing Quantitative Methods for Analyzing Sediment DNA Records of Cyanobacteria in Experimental and Reference Lakes
Source: Front Microbiol. 2021 Jun 18;12:669910. doi: 10.3389/fmicb.2021.669910 (PMC8250803; doi:10.3389/fmicb.2021.669910)
Supplement: Supplementary file 6 [file Image_6.PDF]

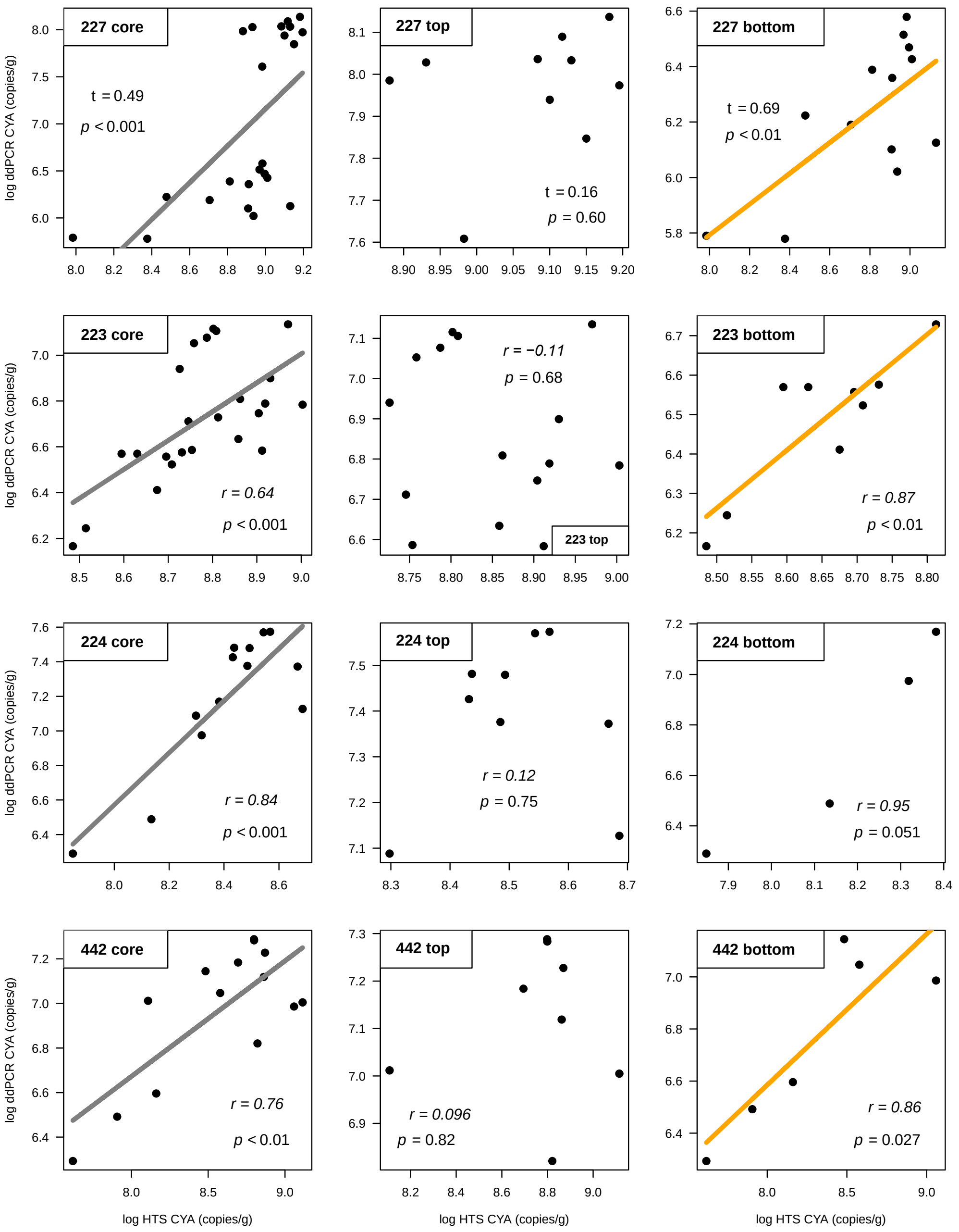

Figure S6. Correlations between log-transformed droplet digital PCR (ddPCR) gene copy numbers and high-throughput sequencing (HTS) amplicon sequence variant counts (using SILVA) for cyanobacterial 16S rRNA (CYA), normalized per gram of wet sediment across the whole core (left), top sediments (middle), and bottom sediments (right) of study lakes. Kendall's τ (Lake 227; top row) and Pearson's *r* (lakes 223, 224, and 442) correlation coefficient values are shown.
