# Supplementary material for: Comparing Quantitative Methods for Analyzing Sediment DNA Records of Cyanobacteria in Experimental and Reference Lakes
Source: Front Microbiol. 2021 Jun 18;12:669910. doi: 10.3389/fmicb.2021.669910 (PMC8250803; doi:10.3389/fmicb.2021.669910)
Supplement: Supplementary file 7 [file Image_7.PDF]

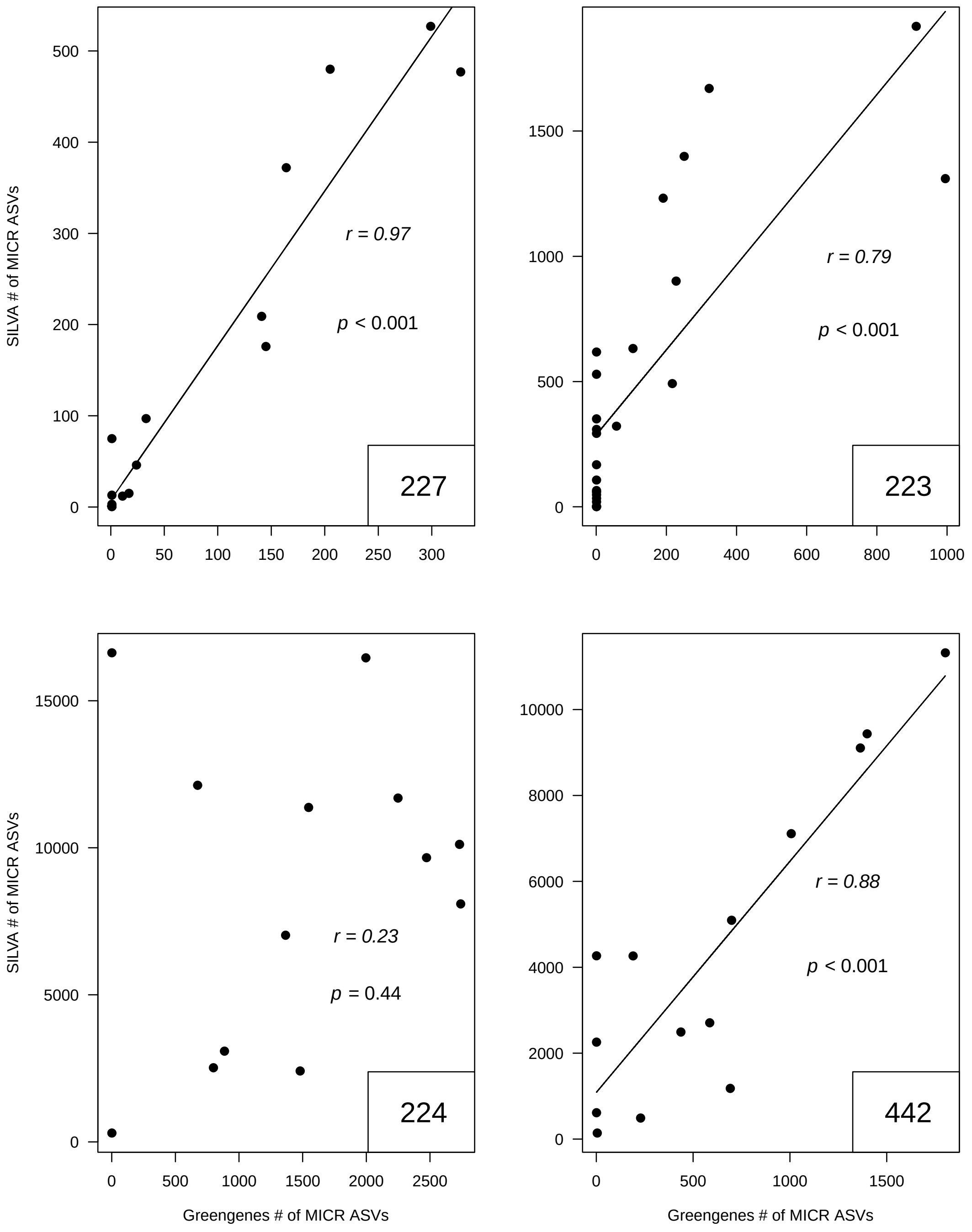

Figure S7. Correlations between SILVA and Greengenes amplicon sequence variant (ASV) counts for *Microcystis* (MICR). Pearson's *r* values are shown.
