# Supplementary material for: Comparing Quantitative Methods for Analyzing Sediment DNA Records of Cyanobacteria in Experimental and Reference Lakes
Source: Front Microbiol. 2021 Jun 18;12:669910. doi: 10.3389/fmicb.2021.669910 (PMC8250803; doi:10.3389/fmicb.2021.669910)
Supplement: Supplementary file 8 [file Image_8.PDF]

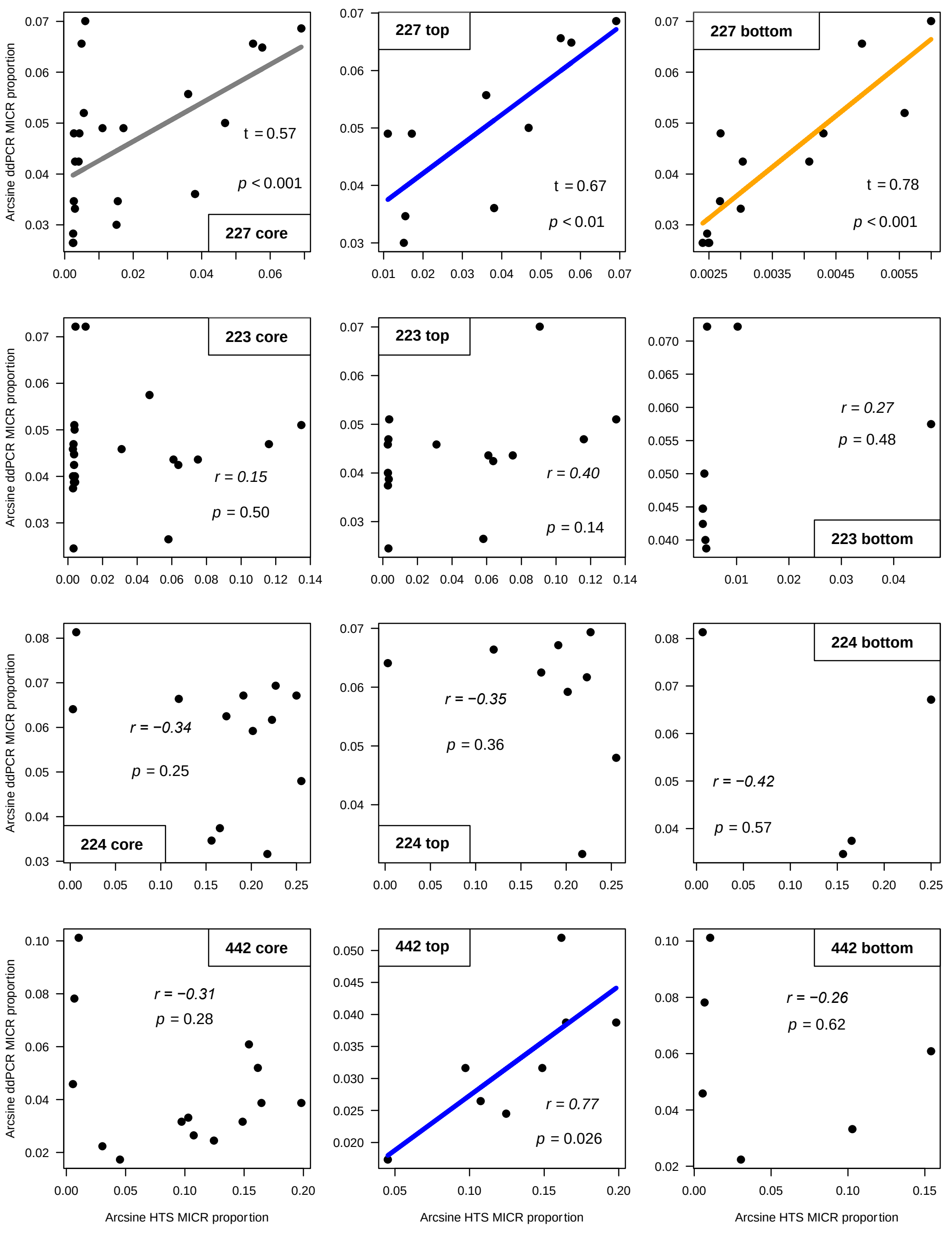

Figure S8. Correlations between arcsine square root transformed droplet digital PCR (ddPCR) and high-throughput sequencing (HTS) (using SILVA) relative abundance outputs for *Microcystis* (MICR) across the whole core (left), top sediments (middle), and bottom sediments (right) of study lakes. Kendall's τ (Lake 227; top row) and Pearson's *r* (lakes 223, 224, and 442) correlation coefficient values are shown.
