# Supplementary material for: Comparing Quantitative Methods for Analyzing Sediment DNA Records of Cyanobacteria in Experimental and Reference Lakes
Source: Front Microbiol. 2021 Jun 18;12:669910. doi: 10.3389/fmicb.2021.669910 (PMC8250803; doi:10.3389/fmicb.2021.669910)
Supplement: Supplementary file 9 [file Table_1.DOCX]

Table S1. Experimental details and morphology* of IISD-ELA study lakes 227, 223, 224, and 442.

| Lake | Coordinates | Lake  type | Manipulation | | Area (ha) | Max depth (m) | Water renewal time (yr) | Source |
| --- | --- | --- | --- | --- | --- | --- | --- | --- |
| 227 | 49°31' N  93°41' W | Experimental —  Eutrophied | Years of experimentation:  N:P^a^ loading ratio:  TN:TP^b^ Concentration (µg/L):  Years of experimentation:  N:P^a^ loading ratio:  TN:TP^b^ Concentration (µg/L):  Years of experimentation:  Nutrient loading:  TN:TP^b^ Concentration (µg/L): | 1969-1974  12:1  ~825:42  1975-1989  4:1  ~1200:42  1990-present  Phosphorus only  ~800:42 | 5.0 | 10.6 | 4 | (Anderson et al., 1987; Schindler et al., 2008) |
| 223 | 49°42' N  93°42' W | Experimental  —  Acidified | Years of experimentation:  H_2_SO_4_ loading:  pH change: | 1976-1983  ~3400 L/year^c^  6.7-5.13 | 27.3 | 14.4 | 8 | (Schindler et al., 1980; Cruikshank, 1984; Findlay and Kasian, 1996) |
| 224 | 49°41' N  93°43' W | Reference | Year of experimentation:  Radioisotope addition:  Year of experimentation:  Radioisotope addition:  Year of experimentation:  Radioisotope addition: | 1976  ^14^C and ^226^Ra  1976  ^75^Se, ^203^Hg, ^137^Cs, ^59^Fe, ^65^Zn, ^60^Co  1976  ^3^H | 25.9 | 27.4 | 13.5 | (Hesslein et al., 1980a; 1980b; Quay et al., 1980; Crusius and Anderson, 1995) |
| 442 | 49°46' N  93°49' W | Reference | NA | | 16.0 | 17.8 | 11.0 | (Higgins et al., 2018) |

*IISD-Experimental Lakes Area (<https://www.iisd.org/ela/science-data/our-data/interactive-map/>)

^a^Nitrogen:Phosphorus^,^ ^b^Total Nitrogen: Total Phosphorus average concentrations in epilimnion during ice-free season, ^c^Average volume added during ice-free season.
