# Supplementary material for: Comparing Quantitative Methods for Analyzing Sediment DNA Records of Cyanobacteria in Experimental and Reference Lakes
Source: Front Microbiol. 2021 Jun 18;12:669910. doi: 10.3389/fmicb.2021.669910 (PMC8250803; doi:10.3389/fmicb.2021.669910)
Supplement: Supplementary file 10 [file Table_2.DOCX]

Table S2. Sediment cores retrieved by IISD-ELA from lakes 227, 223, 224, and 442.

| **Lake** | Date of collection | Core length (cm) | Depth collection (m) | Collected by |
| --- | --- | --- | --- | --- |
| **227** | March 20, 2018 | 55 | 10.6 | Cyndy Desjardins, Paul Faford, Michael Paterson, Stephen Paterson |
| **223** | March 21, 2018 | 55 | 14.60 | Cyndy Desjardins, Stephen Paterson |
| **224** | March 21, 2018 | 37 | 26.2 | Cyndy Desjardins, Stephen Paterson |
| **442** | March 22, 2018 | 55 | 17.2 | Cyndy Desjardins, Paul Faford, Stephen Paterson |
