# Supplementary material for: Comparing Quantitative Methods for Analyzing Sediment DNA Records of Cyanobacteria in Experimental and Reference Lakes
Source: Front Microbiol. 2021 Jun 18;12:669910. doi: 10.3389/fmicb.2021.669910 (PMC8250803; doi:10.3389/fmicb.2021.669910)
Supplement: Supplementary file 11 [file Table_3.DOCX]

Table S3. The range in water content (%) in top and bottom subsection intervals of sediment cores collected from ELA Lake 227, 223, 224, and 442.

| **Lake** | Core Depth (cm) | Range in water content (%) |
| --- | --- | --- |
| **227** | Top (1-15.25) | 96.95 – 98.74 |
|  | Bottom (16.25-51.75) | 95.58 – 96.75 |
| **223** | Top (1-20.25) | 94.85 – 98.79 |
|  | Bottom (21.75-41.75) | 93.48 – 96.04 |
| **224** | Top (1-7.25) | 97.55 – 98.76 |
|  | Bottom (16.25-32.25) | 90.15 – 96.75 |
| **442** | Top (1-12.25) | 95.19 – 99.24 |
|  | Bottom (13.25-39.75) | 77.41 – 96.02 |
