# Supplementary material for: Comparing Quantitative Methods for Analyzing Sediment DNA Records of Cyanobacteria in Experimental and Reference Lakes
Source: Front Microbiol. 2021 Jun 18;12:669910. doi: 10.3389/fmicb.2021.669910 (PMC8250803; doi:10.3389/fmicb.2021.669910)
Supplement: Supplementary file 12 [file Table_4.DOCX]

Table S4. Core depths and associated timeframes from top and bottom sediments from IISD-ELA lakes 227, 223, 224, and 442, based on the ^210^Pb and ^137^Cs radioisotope dating.

|  | **Top sediments (post-manipulation*)** | | **Bottom sediments (pre-manipulation*)** | |
| --- | --- | --- | --- | --- |
| **Lake** | Depth (cm) | Timeframe | Depth (cm) | Timeframe |
| L227 | 0 – 15.25 | 1968 – 2018 | 16.25 – 51.75 | Pre-1850 – 1967 |
| L223 | 0 – 20.25 | 1976 – 2018 | 21.75 – 41.75 | Pre-1850 – 1973 |
| L224 | 0 – 7.25 | 2004 – 2018 | 16.25 – 32.25 | Pre-1850 – 1985 |
| L442 | 0 – 12.25 | 1990 – 2018 | 13.25 – 39.75 | Pre-1850 – 1989 |

*For lakes 227 and 223
